# Prospective memory in the developmental age: a systematic review to synthesize the evaluation tools and the main cognitive functions involved

**DOI:** 10.3389/fpsyg.2024.1394586

**Published:** 2024-10-16

**Authors:** Mariarosaria Guzzardi, Deny Menghini, Floriana Costanzo, Stefano Vicari, Francesca Foti

**Affiliations:** ^1^Department of Health Sciences, “Magna Graecia” University of Catanzaro, Catanzaro, Italy; ^2^Child and Adolescent Neuropsychiatry Unit, Bambino Gesù Children's Hospital, IRCCS, Rome, Italy; ^3^Department of Life Sciences and Public Health, Catholic University of the Sacred Heart, Rome, Italy; ^4^Department of Educational Sciences, University of Catania, Catania, Italy

**Keywords:** prospective memory, ongoing task, event-based prospective memory, time-based prospective memory, school-aged children, schoolchildren, executive functions, development

## Abstract

Prospective memory (PM) is the ability to remember and realize one’s intentions in the future; therefore, it is crucial for the daily functioning of children and adolescents and their ability to become independent from caregivers. PM errors can have repercussions during childhood, such as influencing school performance and social relationships. The aim of this systematic review was to synthesize studies analysing PM in children and adolescents (age range: 0–16 years) following PRISMA guidelines. The goal was to outline the most commonly used tasks, offering information on the development of PM, and—through a detailed analysis of the assessment of specific cognitive processes carried out in the primary studies included—providing information on the main cognitive processes involved in PM within this age group. Forty-nine studies were selected that examined PM in children and adolescents with typical development. The studies used many different tasks that can be traced back to eleven different main paradigms to evaluate PM, each structured into a PM and an ongoing task. Older children performed better on PM targets than younger children, suggesting a developmental trajectory of PM that follows a J-shaped function. Children as young as 2 years old exhibited the first signs of PM, while adolescents performed similarly to adults on PM tasks. Several factors are involved in PM development: retrospective memory, executive functions (planning, working memory, inhibitory control, monitoring), attention, metamemory, and motivation. This review May be considered a starting point to summarize the most used tools to evaluate PM in children and adolescents, and to shed light on the primary cognitive functions involved in PM, potentially offering indications to researchers in selecting optimal tasks for measuring PM across different age groups. Additionally, it underscores the importance of developing standardized measures for potential clinical applications.

## 1 Introduction

One of the most frequent memory challenges in daily life is remembering to remember. Memory in everyday life is defined as perspective memory (Einstein and McDaniel, 1990). While retrospective memory refers to the past, prospective memory (PM) is the capability to remember to carry out one’s intentions in the future; it is critical for children’s daily functioning and their ability to become independent from caregivers (Mahy et al., 2014a). PM can be differentiated into time-based PM and event-based PM. To exemplify, meeting friends in a park at 10 a.m. on Sunday morning is a task based on time, a time-based PM, while remembering to deliver a teacher’s message to parents is a task based on the event, an event-based PM (Yang et al., 2011). Importantly, schoolchildren are often required to remember pre-programmed intentions and implement them in the appropriate context while engaged in an ongoing activity (Cheie et al., 2021). Daily PM errors in children can have several repercussions; for example, these errors can affect school performance, or can create danger, as in forgetting to wear a helmet when cycling. Other consequences can be found in social relationships, and failures of the PM mainly impact interpersonal relations, such as failing to bring a gift to a friend at a birthday party (Brandimonte et al., 2010).

**Figure 1:**
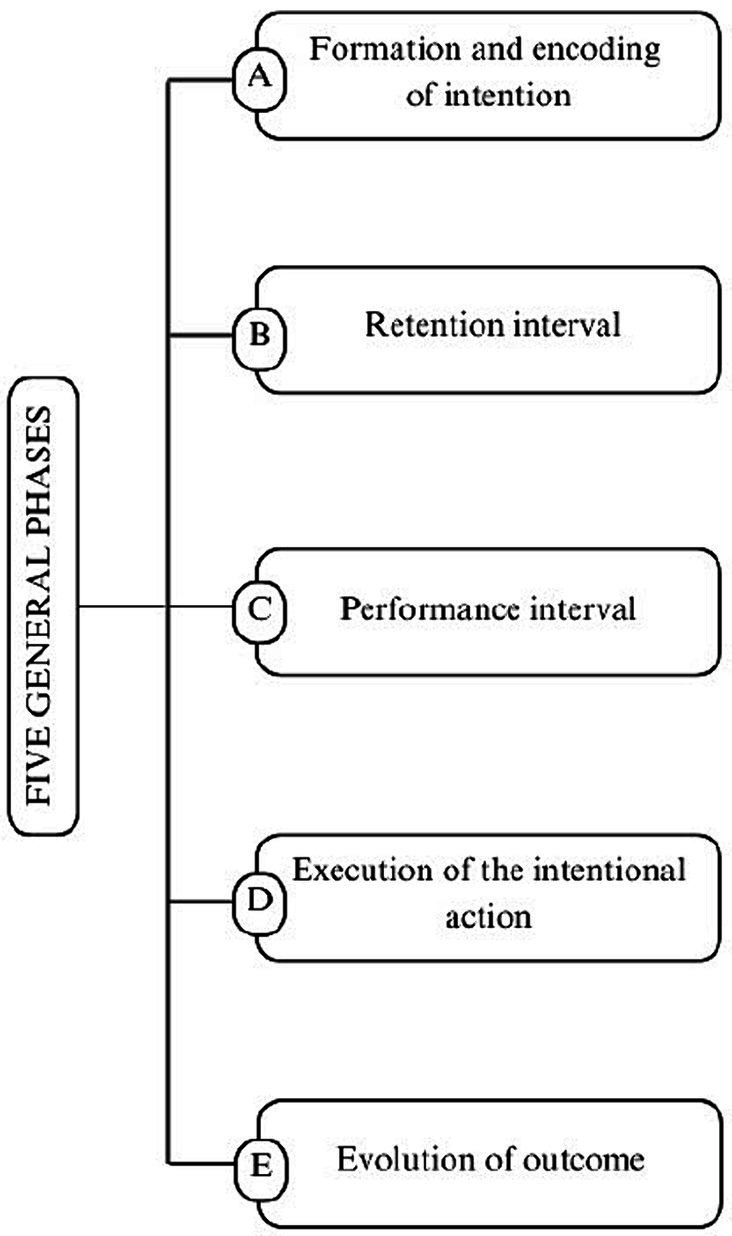
The five general phases that characterize the realization of a future intention.

The realization of future intention is described in five general phases: A. *formation and encoding of intention and action,* mainly concerns the preservation of the content of a delayed intention; B. *retention interval,* refers to the delay between encoding and the beginning of a performance range potential; C. *performance interval,* refers to the performance range or the period during which the intended action is to be recovered; D. *initiation and execution of the intended action* and E. *evolution of outcome,* which concern the initiation and implementation of a planned action and the assessment of the outcome, respectively (Brandimonte et al., 2014) (Figure 1).

PM development follows an inverted U-shape function; in fact, life-long studies show an increase in PM performance during childhood, followed by a peak in early adulthood and a decrease in middle and senior age (Kliegel et al., 2008; Mahy and Moses, 2011; Zimmermann and Meier, 2006). From a neuroanatomical point of view, the studies carried out with functional Magnetic Resonance Imaging (fMRI) have highlighted the activation of the anterior prefrontal cortex during the whole process of prospective memory (Burgess et al., 2001); the frontal (particularly the BA10) and the parietal lobes are also activated during the different processes of perspective memory. These areas are supposed to monitor the intentions in memory and enable to stay focused on them without allowing the accompanying tasks and stimuli to compromise their execution (Burgess et al., 2003).

Regarding the development of PM in children, as far as we know, the difficulty remains in tracing a precise developmental curve, probably due to the absence of a single, valid, and reliable protocol (task) to be able to evaluate PM in developmental age. In general, the tasks used to evaluate PM in children are characterized by two types of activity: a PM task and an ongoing task (OT) (Einstein and McDaniel, 1990). The OT provides a context for the PM action and fills the intervals between the appearances of potential target events. The PM activities consist of monitoring the time (time-based PM task) or remembering to perform a move in the presence of target events (event-based PM task).

### 1.1 Cognitive processes involved in the development of prospective memory

Several factors contribute to the broad trajectory of development of PM from early childhood until adolescence (Mahy et al., 2014a), such as retrospective memory, executive functions (planning, working memory, inhibitory control, monitoring), attention, metamemory, and motivation.

#### 1.1.1 Retrospective memory

The processes of retrospective memory are essential for the smooth functioning of PM, as individuals must remember *what* they need to do and *when* to do it. Many researchers suggest that a successful PM combines two different cognitive processes: a retrospective component, to retain the content of the intention, and an executive processes, that acts at the appropriate time (Ellis, 1996; Kliegel et al., 2002; Mahy et al., 2014a; McDaniel et al., 1999; Smith et al., 2010; Zöllig et al., 2007). There is an agreement in the PM literature that underlines how prospective and retrospective processing components facilitate the realization of delayed intentions (Einstein et al., 1992; Guynn et al., 2001; Simons et al., 2006).

#### 1.1.2 Executive functions

Executive functions (EF) are another significant driving force in the development of PM (Mahy et al., 2014a). There are different EF that are considered central to the development of PM: the shifting between tasks or mental sets (“*Shifting*”); the updating and the monitoring of working memory representations (“*Updating*”); the inhibition of prepotent responses in inappropriate content (“*Inhibition*”) (Mahy et al., 2014a; Mahy and Moses, 2011; Miyake et al., 2000). Specifically, monitoring has a crucial role in PM, and it can be both external and internal. Regarding the first, the environment must be monitored for the appearance of a PM signal. Regarding the second, individuals must also internally monitor their intentions to remember the content of their purpose, and realize it at the appropriate time (Mahy et al., 2014a).

#### 1.1.3 Attention

Norman and Shallice (1986) proposed the theory of the attention system, in which the completion of PM tasks requires attentional resources, and the difficulty of an OT directly affects PM performance (Khan et al., 2008). OT and PM compete for attentional resources, decreasing PM performance (Han et al., 2017). An increase in the difficulty of an OT reduces the available attentional resources that can be assigned to a PM task, negatively affecting its performance (Bisiacchi et al., 2008; Han et al., 2017; Mahy et al., 2015). The effect of OT absorption on PM performance was analysed in children aged 9–10 years and 6–7 years; the results revealed that providing a less demanding OT resulted in better PM performance (Kliegel et al., 2013). Older children performed better in PM tasks because of increased attentive ability, while younger children had more limited attentional resources (Han et al., 2017; Mahy et al., 2015).

#### 1.1.4 Metamemory

Metamemory emerges around 4 to 5 years old, and significantly improves across childhood and into adulthood (Godfrey et al., 2023; Schneider and Lockl, 2008). The knowledge of memory strategies could promote better PM performance. Several studies have shown that knowing about memory functioning (for instance the knowledge of metamemory) can improve people’s memory performance by having them implement appropriate strategies (Hutchens et al., 2012; Lachman and Andreoletti, 2006; McNamara and Scott, 2001). Once the repertoire of strategic metamemory is well stocked with knowledge, and memory skills are well developed, children are more likely to employ one or more strategies to effectively increase their memory performance (DeMarie et al., 2004).

#### 1.1.5 Motivation

Motivation to perform a task significantly impacts children’s performance (Carlson et al., 2005; Hongwanishkul et al., 2005; Kerr and Zelazo, 2004), and PM is no exception to this rule. Higher levels of intrinsic motivation improve children’s intention to perform PM tasks. In contrast, providing small incentives to remember trivial intentions does not seem to affect the performance of PM (Mahy et al., 2014a).

### 1.2 The present systematic review

This systematic review mainly aimed at identifying and describing the tasks used to evaluate PM in childhood and adolescents (age range: 0–16 years), potentially offering indications to researchers in selecting optimal tasks for measuring PM across different age groups. Moreover, this review aimed to provide a general overview of the development of PM in this age group, and to increase the knowledge on the cognitive processes involved in PM, through a detailed analysis of the assessment of specific cognitive functions carried out in the primary studies included.

## 2 Methods

### 2.1 Search strategy and study selection

This systematic review was conducted by searching three databases (Scopus, PsycArticles, and Cochrane Library) to identify articles about PM tasks in children and adolescents with typical development, using the keywords: “Prospective Memory” AND “PM tasks,” AND “Children” OR “Adolescent*” OR “Adolescence.” The search was conducted in December 2023 and updated in July 2024.

### 2.2 Inclusion and exclusion criteria

There were different inclusion and exclusion criteria for eligible studies. First, the target population had to be healthy children and adolescents without any cognitive impairments. Studies with participants who had neurological and psychiatric diagnoses, such as attention-deficit hyperactivity disorder, autism spectrum disorder, learning disabilities, brain injury, epilepsy, depression disorders, anxiety disorders, or significant visual or hearing impairment, were not included. Second, the age of participants had to be between 0 and 16 years.

### 2.3 Screening and study selection with Rayyan

The software Rayyan was used for study selection. The following steps were performed: import of search results into the Rayyan software; screening of titles and abstracts to identify potentially relevant studies; and full-text evaluation to confirm their inclusion.

### 2.4 Data extraction

Data were extracted after determining inclusion and exclusion criteria and selecting the articles to include in the present systematic review. Specifically, the following data were extracted: category of the PM task used; first author and year of publication; specific name of the task used; type of PM evaluated; type of task; other cognitive processes assessed; age range of participants; age of the groups (mean ± SD) and sample size; main results (Table 1).

**Table 1:** Characteristics of studies included in the systematic review.

Task	First author and year	Task name	Type of PM evaluated	Type of task	Other cognitive processes assessed	Age range	Group age (mean ± SD); sample size	Main results	SQACERP
Card Sorting Game	Cejudo et al. (2019a)	–	Event-based	Computerised laboratory PM task	Attention	6–11	Group 1: 6-year-olds (6.88 ± 0.29); 45 participants Group 2: 11-year-olds (11 ± 0.39); 50 participants Total sample size: 95	Older children performed better than younger children in PM task. This finding was related to EF development.	100
	Cejudo et al. (2022)	–	Event-based	Computerised laboratory PM task	Attention, EF	7–11	Group 1: 7-year-olds (7.80 ± 0.32); 14 participants Group 2: 10-year-olds (10.65 ± 1.05); 17 participants Total sample size: 31	Older children performed better at detecting PM stimuli than younger children. In addition, older children could adapt monitoring strategies and the allocation of attention to the tasks’ needs. The ability to regulate attentional strategies, monitoring, and recovery, developed during childhood and affected the performance of PM in situations of attentive difficulty.	95.45
	Ford et al. (2012)	Picture-naming PM test	Event-based	Laboratory PM task	EF	4–6	*Study 1* Total sample size: 59 (5.05 ± 0.5) *Study 2* Total sample size: 50 (4.78 ± 0.5)	A relationship between PM and chronological age was identified as one of the indicators of the development process. EF, particularly inhibition, were important in the development of PM.	100
	Kvavilashvili et al. (2001)	–	Event-based	Laboratory PM task	RM	4–7	*Experiment 1* Group 1: 5-year-olds; 12 participants Group 2: 7-year-olds; 12 participants Total sample size: 24 *Experiment 2* Group 1: 4-year-olds; 20 participants Group 2: 5-year-olds; 20 participants Group 3: 7-year-olds; 20 participants Total sample size: 60 *Experiment 3* Group 1: 4-year-olds; 16 participants Group 2: 5-year-olds; 16 participants Group 3: 7-year-olds; 16 participants Total sample size: 48	A significant difference between preschool and school age children was identified; older children performed better than younger ones. No relationship was found between performance on PM and RM activities.	100
	Kvavilashvili and Ford (2014)	–	Event-based Time-based	Laboratory PM task	MM, RM	5	Study 1: 5-year-olds (5.5); 46 participants Study 2: 5-year-olds (5.4); 80 participants Study 3: 5-year-olds (5.6); 35 participants	5-year-olds demonstrated a remarkable ability to predict the results of PM tasks. MM proved to be crucial in the development of PM; performance predictions stimulated participants’ engagement in PM management or enhanced the activation of their plan, making it more accessible for goal attainment. PM and RM functioned independently in this young age group.	95.45
	Kretschmer-Trendowicz et al. (2016)	–	Event-based	Laboratory PM task	Attention	5–7	Group 1: 5-year-olds (5.52 ± 0.27); 41 participants Group 2: 7-year-olds (7.36 ± 0.26); 39 participants Total sample size: 80	School-age children significantly outperformed preschool children. RM, particularly episodic future thinking strategies, improved children’s PM performance.	100
	Lavis and Mahy (2021)	PM card sorting task	Event-based	Laboratory PM task	EF, MM	4–6	Group 1: 4-year-olds (4.5 ± 0.28); 47 participants Group 2: 5-year-olds (5.5 ± 0.28); 41 participants Group 3: 6-year-olds (6.5 ± 0.30); 43 participants Total sample size: 131	Performance in children’s PM improved with age. MM judgments developed during preschool, although a significant relationship was found between PM and MM quite early in development.	100
	Mahy and Moses (2011)	Card-sorting game	Event-based	Laboratory PM task	EF	4–6	Group 1: 4-year-olds (4.41 ± 0.45); 32 participants Group 2: 5-year-olds (5.46 ± 0.34); 32 participants Group 3: 6-year-olds (6.47 ± 0.32); 32 participants. Total sample size: 96	Performance in children’s PM improved with age. EF, particularly WM, allowed for an integrative understanding of many of the processes involved in children’s PM.	100
	Mahy et al. (2014b)	Card Sort	Event-based	Laboratory PM task	EF	4–5	Group 1: 4-year-olds (4.41 ± 0.29); 56 participants Group 2: 5-year-olds (5.51 ± 0.32); 56 participants Total sample size: 112	Older children outperformed younger children. EF, particularly inhibition, had an important role on PM performance.	100
	Mahy and Moses (2015)	Card-sorting game	Event-based	Laboratory PM task	EF	4–5	Group 1: 4-year-olds (4.4 ± 0.28); 32 participants Group 2: 5-year-olds (5.6 ± 0.33); 32 participants Total sample size: 64	Performance in children’s PM improved with age. EF, especially monitoring, played a critical role in PM development.	100
	Mahy et al. (2016)	Card-sorting game	Event-based	Laboratory PM task	EF	4–5	Group 1: 4-year-olds (4.39 ± 0.26); 32 participants Group 2: 5-year-olds (5.5 ± 0.34); 32 participants Total sample size: 64	Younger children showed worse performance in PM than older children. EF, particularly WM, were notably correlated with PM performance.	100
	Ryder et al. (2022)	-	Event-based	Laboratory PM task	-	5–7	Group 1: 5-year-olds (5.36 ± 0.26); 80 participants Group 2: 7-year-olds (7.40 ± 0.26); 80 participants Total sample size: 160	Older children significantly outperformed younger children in the PM task.	95.45
	Szpakiewicz and Stępień-Nycz (2024)	Card recognition test	Event-based	Laboratory PM task	EF	2–6	Group 1: 2 years (2.7 ± 0.6); 36 participants Group 2: 3 years (3.5 ± 0.6); 39 participants Group 3: 4 years (4.5 ± 0.6); 47 participants Group 4: 5 years (5.5 ± 0.6); 50 participants Group 5: 6 years (6.5 ± 0.5); 52 participants Total sample size: 224	Very young children can successfully solve PM tasks. Accuracy in PM tasks improved with age, especially between 3 and 5 years. EF—such as inhibitory control, WM, and cognitive monitoring—correlated with PM performance.	100
	Wang et al. (2008)	-	Event-based	Laboratory PM task	EF, RM	3–5	Experiment 1: Group 1: 3-year-olds (3.10 ± 0.64); 20 participants Group 2: 4-year-olds (4.56 ± 0.51); 19 participants Group 3: 5-year-olds (5.24 ± 0.44); 21 participants Total sample size: 60 Experiment 2: Group 1: 3-year-olds (3.40 ± 0.50); 20 participants Group 2: 4-year-olds (4.50 ± 0.51); 22 participants Group 3: 5-year-olds (5.15 ± 0.37); 20 participants Total sample size: 62	Older children showed better PM performance. EF, mainly inhibitory control, appeared to be an influential factor for PM task performance at developmental age. RM influenced PM response time, but not PM accuracy.	100
	Zhang et al. (2017)	Card-naming task	Event-based	Laboratory PM task	EF, motivation, RM	3–5	Group 1: 3-4-year-olds (3.651 ± 0.22); 40 participants Group 2: 5-year-olds (5.58 ± 0.26); 40 participants Total sample size: 80	PM performance was significantly higher in older children than in younger children. EF, particularly monitoring, were crucial strategies for PM tasks, suggesting that the development of executive functioning leads to increased prospective abilities during early childhood. Motivation played an essential role in the success of PM tasks. RM was necessary to remember the deliberate intention.	100
	Zhang et al. (2019)	-	Event-based	Laboratory PM task	EF	3–5	Group 1: 4-year-olds (4.41 ± 0.45); 32 participants Group 2: 5-year-olds (5.46 ± 0.34); 32 participants Group 3: 6-year-olds (6.47 ± 0.32); 32 participants Total sample size: 96	Older children showed better performance in PM than younger children.	100
	Zuber et al. (2019)	Size Sorting Task	Event-based	Computerised laboratory PM task	EF	6–11	Group 1: 6-year-olds (6.2 ± 0.3); 26 participants Group 2: 7-year-olds (7.0 ± 0.4); 55 participants Group 3: 8-year-olds (8.0 ± 0.4); 40 participants Group 4: 9-year-olds (9.1 ± 0.3); 43 participants Group 5: 10-year-olds (10.2 ± 0.3); 36 participants Group 6: 11-year-olds (10.10 ± 0.3); 12 participants Total sample size: 212	School-age children showed better performance in PM than preschool age children. EF—especially updating, shifting, inhibition, and monitoring—contributed to PM development.	100
Picture Classification task	Basso et al. (2023)	-	Event-based	Semi-ecological laboratory PM computer task	EF	8–12	Group 1: 8-year-olds (8.12 ± 0.44); 76 participants Group 2: 12-year-olds (12.21 ± 0,53); 82 participants Total sample size: 158	Older children were more efficient in performing the PM task than younger children, showing greater speed without significant differences in accuracy. A significant effect of WM emerged only in the younger children group. No effect of inhibition was observed in younger and older groups.	100
	Chen et al. (2017)	-	Event-based	Semi-ecological laboratory PM computer task	EF	13	Group 1: 13-year-olds (13.61 ± 1.86); 59 participants Group 2: 13-year-olds (13.37 ± 1.79); 54 participants Total sample size:103	EF were crucial in PM performance, especially in initial coding, maintenance, and intention retrieval.	100
	Cottini et al. (2018)	Picture classification task	Event-based	Semi-ecological laboratory PM computer task	EF, MM	7	Group 1: 7-year-olds (7.42 ± 0.29); 30 participants Group 2: 7-year-olds (7.32 ± 0.25); 29 participants Total sample size: 59	School-age children used MM strategies—making performance predictions improved performance in PM. EF, particularly monitoring and WM, were essential to children’s PM success.	100
	Cottini et al. (2019)	Picture classification task	Event-based	Semi-ecological laboratory PM computer task	EF, MM	7–8	Group 1: 7-8-year-olds (7.81 ± 0.34); 31 participants Group 2: 7-8-year-olds (7.79 ± 0.25); 32 participants Total sample size: 63	PM underwent significant developmental changes during primary school years. EF, especially monitoring, were important in children’s PM. MM was an essential element in the development of PM; school-age children had a good understanding of their PM abilities.	100
	Cottini et al. (2021b)	Picture classification task	Event-based	Semi-ecological laboratory PM computer task	EF, MM	5–10	Group 1: 5-6-year-olds (5.7 ± 0.3); 49 participants Group 2: 8-10-year-olds (9.5 ± 0.12); 35 participants Total sample size: 84	Older children outperformed younger children in PM. EF, particularly monitoring, improved with age, supporting PM. MM was crucial in PM development, as older children’s predictions and post-diction were closer to actual PM performance than younger children’s.	100
	Cottini et al. (2021a)	Picture classification task	Event-based	Semi-ecological laboratory PM computer task	EF, MM	7–11	Group 1: 7–11 year-olds (9.8 ± 0.11); 32 participants Group 2: 7-11-year-olds (9.6 ± 0.11); 37 participants Group 3: 7-11-year-olds (9.5 ± 0.11); 32 participants Group 4: 7-11-years-olds (9.7 ± 0.11) 26 participants Total sample size: 127	MM significantly improved children’s PM performance, mainly using predictions as strategies.	100
	Guajardo and Best (2000)	Computer task	Event-based	Semi-ecological laboratory PM computer task	EF, motivation, RM	3–5	Group 1: 3-year-olds (M = 3.5); 48 participants Group 2: 5-year-olds (M = 5.25); 48 participants Total sample size: 96	Older children showed better PM performance than younger children. Differences in the development of EF, particularly WM, could also explain differences in PM performance in preschoolers. Central executive processing was critical for the successful completion of PM tasks. Motivating children with rewards could improve children’s performance on PM tasks. PM and RM performance of younger children were correlated.	100
	Han et al. (2017)	-	Event-based Time-based	Laboratory PM task	Attention, motivation	3–5	Experiment 1 Group 1: 3-year-olds (3.33 ± 0.0.22); 38 participants Group 2: 4-year-olds (4.27 ± 0.23); 32 participants Group 3: 5-year-olds (5.22 ± 0.23); 35 participants Total sample size: 105 Experiment 2 Group 1: 5-year-olds Group 2: 5-year-olds Total sample size: 103 (5.20 ± 0.16) Experiment 3 Group 1: 5-year-olds Group 2: 5-year-olds Total sample size: 106 (5.25 ± 0.21)	School-age children outperformed preschool-age children. Attention played an essential role in children’s PM tasks.	100
	Hartwig et al. (2021)	-	Event-based	Computerised laboratory PM task	EF	6–10	Group 1: 6-7-year-olds (6.7 ± 0.5); 46 participants Group 2: 9-10-year-olds (9.4 ± 0.6); 45 participants Total sample size: 91	PM developed during childhood; older children outperformed younger children in PM tasks. EF, especially WM, significantly affected children’s PM performance.	100
	Kretschmer-Trendowicz et al. (2021)	-	Event-based	Computerised laboratory PM task	EF	9–16	Experiment 1 Group 1: 9-10-year-olds (9.10 ± 0.0.31); 30 participants Group 2: 11-13-year-olds (12.17 ± 0.48); 47 participants Group 3: 14-16-year-olds (15.06 ± 0.39); 47 participants Total sample size: 124 Experiment 2 Group 1: 8-10-year-olds (9.00 ± 0.0.33); 37 participants Group 2: 11-13-year-olds; 46 participants Group 3: 14-16-year-olds; 46 participants	PM performance increased significantly from childhood to adolescence. Improvements in PM were associated with EF, particularly WM and shifting. RM helped the success of prospective action.	100
	Walsh et al. (2014)	The Shopping Trip	Event-based	Semi-ecological laboratory PM computer task	EF, MM, RM	3–5	Experiment 1: Group 1: 3-year-olds (3.37 ± 0.30); 25 participants Group 2: 4-year-olds (4.46 ± 0.27); 27 participants Group 3: 5-year-olds (5.44 ± 0.33); 19 participants Total sample size: 71 Experiment 2: Group 1: 3-year-olds (3.47 ± 0.37); 23 participants Group 2: 4-year-olds (4.54 ± 0.34); 16 participants Group 3: 5-year-olds (5.70 ± 0.45); 17 participants Total sample size: 56	PM performance in children improved with age. PM and RM followed the same developmental trajectory. RM favoured the successful performance of PM.	100
	Wang et al. (2024)	Time-Based Prospective Memory Task	Time-based	Computerised laboratory PM task	EF	7–11	Experiment 1 Group 1: 7-year-olds (7.65 ± 0.69); 40 participants Group 2: 9-year-olds (9.3 ± 0.51); 40 participants Group 3: 11-year-olds (10.63 ± 0.62) 40 participants Total sample size: 120 Experiment 2 Group 1: 7-year-olds (7.59 ± 0.76); 61 participants Group 2: 9-year-olds (9.22 ± 0.48); 60 participants Group 3: 11-year-olds (10.56 ± 0.63) 60 participants Total sample size: 181	PM developed between the ages of seven and eleven, with marked improvements from the age of nine. EF, especially time monitoring, developed with age: older children used more effective monitoring strategies than younger children. Attention contributed to the maturation of PM.	100
Cyber Cruiser	Cheie et al. (2021)	Cybercruiser-II	Time-based	Computerised laboratory PM task	EF, RM	6–10	Group 1: 6-7-year-olds (6.76 ± 0.44); 33 participants Group 2: 8-9-year-olds (8.59 ± 0.50); 29 participants Group 3: 10-year-olds (10.32 ± 0.14); 20 participants Total sample size: 82	Performance increased with age. EF—especially updating, inhibition, displacement, and monitoring—improved PM performance.	100
	Kerns (2000)	CyberCruiser	Time-based	Computerised laboratory PM task	EF	7–12	Total sample size: 80 (10.03 ± 1.72)	Older children showed better PM performance than younger children. EF—particularly WM, inhibitory control, monitoring, and planning—allowed for fewer errors in PM tasks.	100
	Kliegel et al. (2013)	Dresden Cruiser	Time-based	Computerised laboratory PM task	EF	6–10	Experiment 1: Group 1: 6-7-year-olds (6.88 ± 0.33); 33 participants Group 2: 9-10-year-olds (9.67 ± 0.54); 33 participants Total sample size: 66 Experiment 2: Group 1: 6-7-year-olds (7.25 ± 0.49); 37 participants Group 2: 9-10-year-olds (9.73 ± 0.51); 39 participants Total sample size: 76 Experiment 3: Group 1: 6-7-year-olds (6.68 ± 0.47); 39 participants Group 2: 9-10-year-olds (9.51 ± 0.51); 39 participants Total sample size: 78	Older children outperformed younger children on PM tasks. EF development guided the development of PM in elementary school age. RM was a valuable component of task instruction.	100
	Mahy et al. (2015)	Dresden Cruiser	Time-based	Computerised laboratory PM task	Attention, EF	5–12	Group 1: 5-year-olds (4.83–6.17); 40 participants Group 2: 7-year-olds (6.83–8.17); 43 participants Group 3: 9-year-olds (8.83–10.17); 43 participants Group 4: 11-year-olds (10.83–12.17); 40 participants Total sample size: 166	Older children showed better PM performance than younger children. Children performed worse in the divided attention condition than in the sustained attention condition. EF, especially monitoring, were critical to PM success.	100
	Voigt et al. (2014)	Dresden Cruiser	Time-based	Computerised laboratory PM task	EF	5–14	Group 1: 5-6-year-olds; 33 participants Group 2: 7-8—year-olds; 39 participants Group 1: 9-10-year-olds; 40 participants Group 2: 11-12—year-olds; 38 participants Group 2: 13-14—year-olds; 27 participants Total sample size: 177 (9.04 ± 2.79)	PM increased linearly from age five to age fourteen. EF—such as WM, shifting and time monitoring—influenced time-based PM. Older participants tended to use more proactive and effective control strategies compared to younger participants.	100
	Voigt et al. (2015)	Dresden Cruiser	Time-based	Computerised laboratory PM task	EF	6–10	Group 1: 6-7-year-olds (7.2 ± 0.55); 27 participants Group 2: 9-10—year-olds (9.61 ± 0.71); 27 participants Total sample size: 54	Older children performed better than younger children. EF, particularly monitoring, were associated with age differences in PM tasks.	100
	Zuber et al. (2019)	Swiss Cruiser	Time-based	Computerised laboratory PM task	EF	6–11	Group 1: 6-year-olds (6.2 ± 0.3); 26 participants Group 2: 7-year-olds (7.0 ± 0.4); 55 participants Group 3: 8-year-olds (8.0 ± 0.4); 40 participants Group 4: 9-year-olds (9.1 ± 0.3); 43 participants Group 5: 10-year-olds (10.2 ± 0.3); 36 participants Group 6: 11-year-olds (10.10 ± 0.3); 12 participants Total sample size: 212	School age children showed better performance in PM than preschool age children. EF—especially updating, shifting, inhibition, and monitoring—contributed to PM development.	100
Virtual Week	Terrett et al. (2019)	Virtual Week	Event-based Time-based	Semi-ecological laboratory PM computer task	RM	8–12	Total sample size: 62	RM, and future episodic thinking, supported PM performance by strengthening the encoding of PM task details.	100
	Yang et al. (2011)	Happy Week	Event-based Time-based	Semi-ecological laboratory PM computer task	EF	7–12	Group 1: 7-year-olds; 20 participants Group 2: 8-year-olds; 20 participants Group 3: 9-year-olds; 20 participants Group 4: 10-year-olds; 20 participants Group 5: 11-year-olds; 20 participants Group 6: 12-year-olds; 20 participants Total sample size: 120	As age increased, children’s accuracy in PM tasks improved. EF, particularly WM and inhibition, were linked to PM.	100
Fishing Game	Cheie et al. (2021)	Fishing Game	Event-based Time-based	Computerised laboratory PM task	EF, RM	6–10	Group 1: 6-7-year-olds (6.76 ± 0.44); 33 participants Group 2: 8-9-year-olds (8.59 ± 0.50); 29 participants Group 3: 10-year-olds (10.32 ± 0.14); 20 participants Total sample size: 82	Performance increased with age. EF—especially updating, inhibition, displacement, and monitoring—improved PM performance.	100
	Yang et al. (2011)	Fishing Game	Event-based Time-based	Computerised laboratory PM task	EF	7–12	Group 1: 7-year-olds; 20 participants Group 2: 8-year-olds; 20 participants Group 3: 9-year-olds; 20 participants Group 4: 10-year-olds; 20 participants Group 5: 11-year-olds; 20 participants Group 6: 12-year-olds; 20 participants Total sample size: 120	Performance increased with age. EF, particularly WM and inhibition, were linked to PM.	100
	Yang et al. (2023)	Fishing Game	Event-based Time-based	Computerised laboratory PM task	EF, MM	7–11	Group 1: 7-11-year-olds (9.04 ± 1,42); 78 participants Group 2: 7-11-year-olds (9.04 ± 1,45); 76 participants Total sample size: 154	Age was a significant predictor for both event-and time-based PM, indicating development during mid-childhood. WM facilitated the retention only in time-based, but not in event-based PM task. MM predicted time-based PM only when there were sufficient cognitive resources/EF. Both EF and MM were crucial for the success of memory strategies.	100
Ask for stickers	Causey and Bjorklund, (2014)	-	Event-based	Semi-ecological laboratory PM task	EF, MM, motivation	2–4	Total sample size: 31	Preschoolers showed good PM if the task was important to them. EF were essential to the development of PM. MM and motivation also played crucial roles in PM development.	95.45
	Guajardo and Best, 2000	Naturalistic task	Event-based	Semi-ecological laboratory PM task	EF, motivation, RM	3–5	Group 1: 3-year-olds (M = 3.5); 48 participants Group 2: 5-year-olds (M = 5.25); 48 participants Total sample size: 96	Older children showed better PM performance than younger children. Differences in the development of EF, particularly WM, could also explain differences in PM performance in preschoolers. Central executive processing was critical for the successful completion of PM tasks. Motivating children with rewards could improve children’s performance on PM tasks. PM and RM performance of younger children were correlated.	100
	Hashimoto et al. (2022)	-	Event-based	Semi-ecological laboratory PM task	Attention, EF	7–15	Total simple size: 94	WM and monitoring positively affected children performance. Moreover, attention was associated with PM success.	100
	Kelly et al. (2023)	-	Event-based	Semi-ecological laboratory PM task	-	4–6	*Experiment 1* Total sample size: 17 participants (5.61 ± 0.35) *Experiment 2* Total sample size: 22 participants (4.5 ± 0.29)	Younger children could quickly learn to perform common PM tasks. The study emphasized that there are practice-related mechanisms, independent of EF, that play a key role in influencing the development and performance of PM.	95.45
	Ślusarczyk and Niedźwieńska (2013)	-	Event-based	Semi-ecological laboratory PM task	EF, MM	2–6	Study 1: Group 1: 2-year-olds (2.7); 23 participants Group 2: 3-year-olds (3.5); 30 participants Group 3: 4-year-olds (4.5); 30 participants Group 4: 5-year-olds (5.3); 30 participants Group 5: 6-year-olds (6.5); 30 partecipants Total sample size: 143 Study 2: Group 1: 3-year-olds (3.58); 44 participants Group 2: 6-year-olds (6.33); 46 participants Total sample size: 90	PM performance improved systematically during preschool years. EF, mainly inhibitory control, played an essential role in PM development. The high motivation was necessary for two-year-olds to perform well, which remained an important factor that increased performance throughout preschool.	100
	Ślusarczyk et al. (2018)	PM task	Event-based	Semi-ecological laboratory PM task	EF, motivation	2	Total sample size: 158	Two-year-olds were successful in PM activities. The motivation was a crucial factor in successful PM performance.	95.45
	Walsh et al. (2014)	Ask for Sticker	Event-based	Semi-ecological laboratory PM task	EF, MM, RM	3–5	Experiment 1: Group 1: 3-year-olds (3.37 ± 0.30); 25 participants Group 2: 4-year-olds (4.46 ± 0.27); 27 participants Group 3: 5-year-olds (5.44 ± 0.33); 19 participants Total sample size: 71 Experiment 2: Group 1: 3-year-olds (3.47 ± 0.37); 23 participants Group 2: 4-year-olds (4.54 ± 0.34); 16 participants Group 3: 5-year-olds (5.70 ± 0.45); 17 participants Total sample size: 56	PM performance in children improved with age. PM and RM followed the same developmental trajectory. The contribution of RM favoured the successful performance of PM.	100
Sightseeing tour	Kretschmer-Trendowicz et al. (2019)	Sightseeing tour	Event-based	Semi-ecological laboratory PM task	RM	10–12	Group 1: 10-12-year-olds (10.82 ± 0.86); 28 participants Group 2: 10-12-year-olds (10.64 ± 0.68); 28 participants Total sample size: 56	PM performance increased in infancy. RM, mainly encoding future episodic thinking as a strategy, helped improve PM.	100
MISTY	Mills et al. (2021)	MISTY	Event-based Time-based	Paper and pencil laboratory PM task	EF, MM, RM	4–15	Group 1: 4-6-year-olds; 21 participants Group 2: 7-8-year-olds; 32 participants Group 3: 9-10-year-olds; 20 participants Group 4: 11-12-years; 25 participants Group 5: 13-15-years 26 participants Total sample size: 124	PM performance significantly increased with age. There was a relationship between PM development and RM, MM, and EF.	100
Mouse task	Geurten et al. (2016)	Mouse task	Time-based	Computerised laboratory PM task	EF, MM	4–9	Group 1: 4-year-olds (4.41 ± 0.45); 24 participants Group 2: 6-year-olds (6.57 ± 0.25); 24 participants Group 3: 9-year-olds (9.47 ± 0.23); 24 participants Total sample size: 72	EF, especially monitoring, positively affected children’s PM performance. Knowledge of MM can be a strategy to improve PM performance during childhood.	100
The Puzzle/Reading Task/Find the Differences/Math Tasks	Cejudo et al. (2019b)	The Puzzle/Reading task/Find the differences/Math problems tasks	Event-based	Laboratory PM task	EF, motivation	6–10	Group 1: 6-7-year-olds (6.89 ± 0.38); 63 participants Group 2: 10-11-year-olds (10.99 ± 0.39); 52 participants Total sample size: 115	Older children performed better than younger children. PM performance during childhood was probably correlated with the development of EF. Motivation played an essential role in PM performance during development.	100
	Shum et al. (2008)	PM task	Event-based	Semi-ecological laboratory PM task	EF	8–13	Group 1: 8-9-year-olds; 35 participants Group 2: 12-13-year-olds; 28 participants Total sample size: 63	PM performance improved with age. EF, especially time monitoring, developed with age: older participants used more effective monitoring strategies than younger participants. Attention contributed to the maturation of PM.	100
Reminding task	Somerville et al. (1983)	Reminding task	Event-based Time-based	Naturalistic task	Motivation, RM	2–4	Group 1: 2-year-olds (2.6); 10 participants Group 2: 3-year-olds (3.41); 10 participants Group 3: 4-year-olds (4.5); 10 participants • Total sample size: 30	Successful recall in PM performance was also evident in younger children. Motivation and high-interest tasks solicited children, promoting successful recall.	95.45

EF, Executive Functions; MM, Metamemory; PM, Prospective Memory; RM, Retrospective Memory; WM, Working Memory; SQACERP, Standard Quality Assessment Criteria for Evaluating Research Papers.

## 3 Results

### 3.1 Selected studies

Research on the different databases identified 1,171 studies. In addition, six other articles were included during the identification phase. These were inserted because they were essential for a more accurate and thorough study of PM tasks. In the screening phase, the results of the three databases were cross-checked, and several duplicates were found. Indeed, twenty-nine duplicate studies were excluded. During this screening phase, the titles and abstracts of the articles were examined for a first selection; fifty-three articles were accepted, and one thousand and ninety-five studies were excluded. Instead, at the eligibility stage, the full text of each article was evaluated. In this step of the PRISMA, four articles for the sample were excluded: one article was excluded because the study sample suffered from anxiety disorders; another article was excluded because it was based on training rather than PM assessment; two other articles were excluded because they presented a sample of adults. Forty-nine articles were assessed as eligible after reading the whole text.

**Figure 2:**
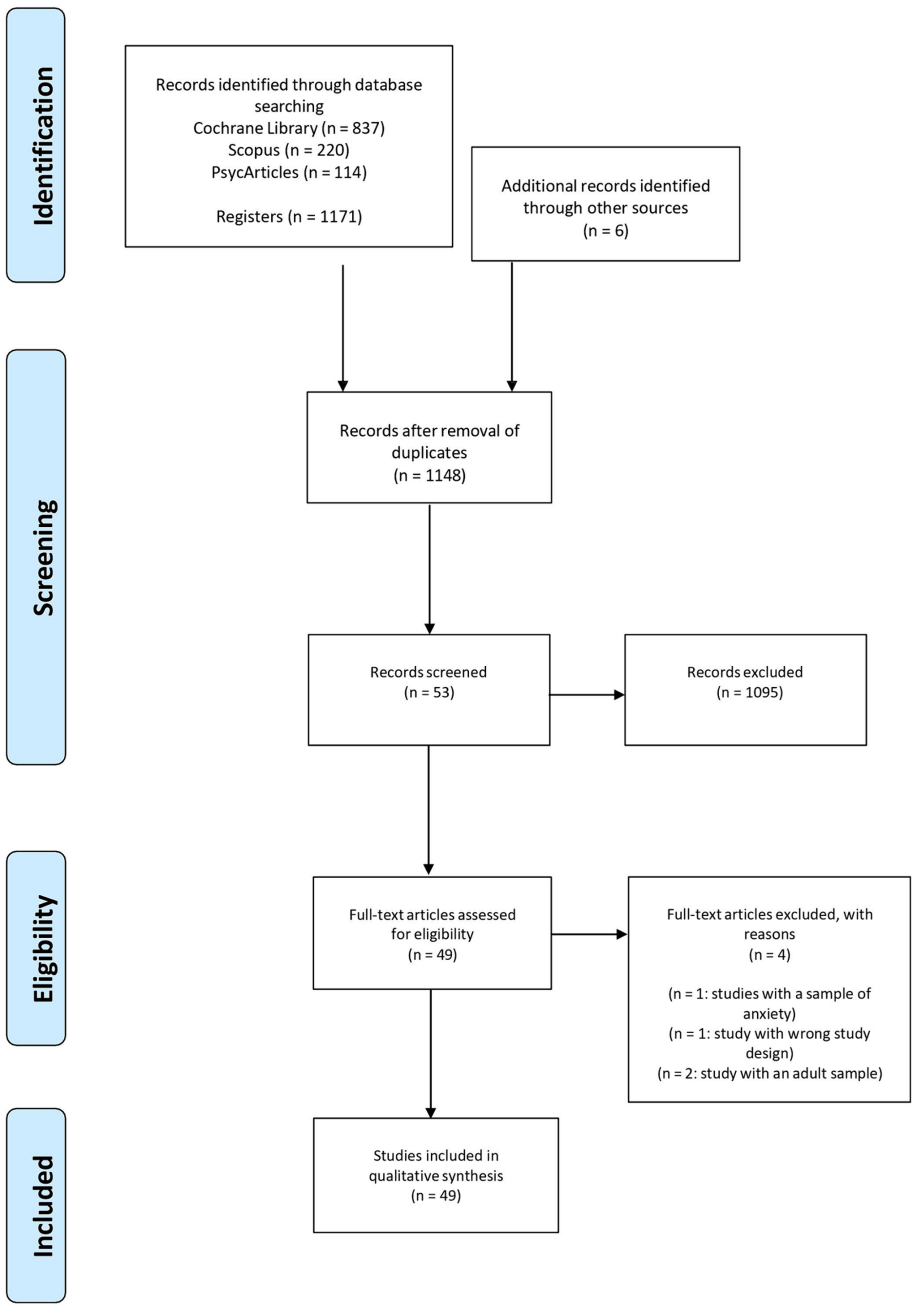
PRISMA flowchart showing the selection of articles included in the systematic review.

A rigorous process of methodological quality assessment was carried out for each study included in the systematic review to ensure that the selected studies met high standards of scientific rigor and that the results were reliable and valid. The assessment was conducted using the “Standard Quality Assessment Criteria for Evaluating Primary Research Papers from a Variety of Fields” (Kmet et al., 2004). These standardized criteria were used to systematically examine and classify each study. As a result of this assessment, only high-quality studies that met the defined standards were included in the final synthesis of the review, which contributed significantly to the overall robustness and credibility of the results. The instrument comprises 14 items to assess methodological bias and error in quantitative and qualitative studies with different study designs. Items that did not apply to a specific study were marked “N/A” and excluded from the calculation of the total score. Each study must be assessed individually to determine which criteria are applicable and which are not. The non-applicable criteria (N/A) should be excluded from the calculation of both the total and maximum score for each study. Specifically, three items (5: randomization, 6: investigator blinding, 7: subject blinding) were removed from QualSyst if not applicable, due to the observational design of the included studies. Each item was scored to indicate whether the study met a criterion (0 = no, 1 = partially, 2 = yes). The scores of the remaining items were summed to create a total score, which was then converted into a percentage (obtained total score divided by the maximum total score). The results were classified as “high quality” (100–90%), “good quality” (89–70%), “moderate quality” (69–50%) and “low quality” (<50%). No study was excluded from the review based on quality alone. Finally, in the inclusion phase, forty-nine studies were selected for this systematic review (see Figure 2; Table 1).

### 3.2 Prospective Memory Tasks

**Figure 3:**
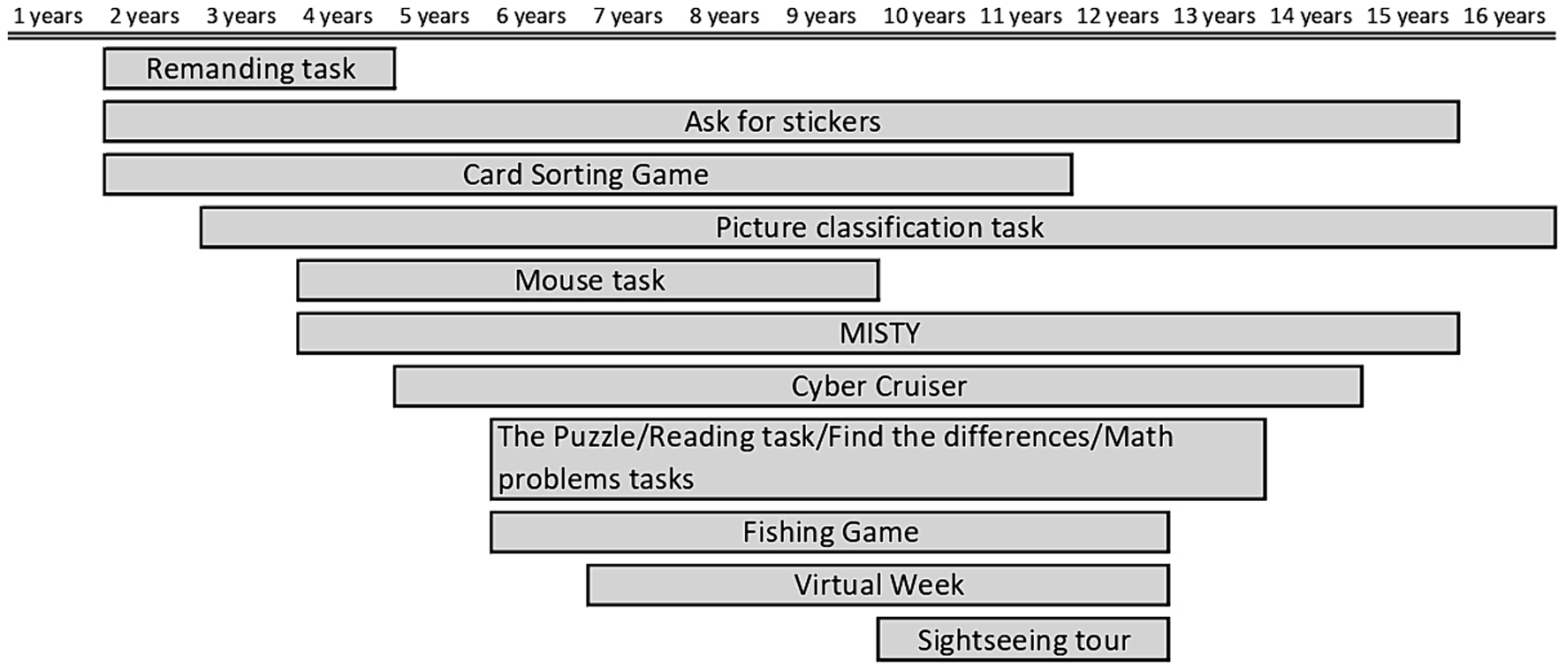
Overview of the eleven main different paradigms employed in the primary studies to evaluate PM, taking into account the participants’ age.

The articles included in the present systematic review evaluated PM using many different tasks that can be traced back to eleven main paradigms, described later in this section (see Table 1). Moreover, we synthetically reported the age in which each paradigm was used in Figure 3. This snapshot could offer indications to researchers regarding the optimal tasks to measure PM in different ages.

#### 3.2.1 Card Sorting Game

The *Card Sorting Game* is a paradigm proposed by Kvavilashvili et al. (2001) used to evaluate event-based PM in the 2 to 11 years age range (Figure 3). The task is presented as a game. Participants sort cards in four decks while naming the objects depicted and respond differently to certain target cards. Specifically, participants must call and sort, in a box, the cards of the deck (OT) while remembering to hide the target cards (PM task).

Different and numerous versions of this paradigm have been proposed. In the *Morris the Mole* version, participants name the objects depicted on the cards, place them face down on a box (OT), and hide the animal card (PM task). Other stimuli used in this version as PM cues are houses (Cejudo et al., 2019a; Ford et al., 2012; Kretschmer-Trendowicz et al., 2016; Kvavilashvili et al., 2001; Kvavilashvili and Ford, 2014; Mahy et al., 2016; Mahy and Moses, 2011, 2015; Szpakiewicz and Stępień-Nycz, 2024; Zhang et al., 2017). In the version adapted by Wang et al. (2008), participants should name the objects depicted on stickers placed on basketball balls (OT) and throw the ball into a basket when the image is an animal (PM task) (Wang et al., 2008). In the version adapted by Mahy et al. (2014b) and Zuber et al. (2019), participants order household items depicted on cards based on two categories: small or large objects (OT). Children ring a bell when they see a depicted animal (PM task) (Mahy et al., 2014b; Zuber et al., 2019). Finally, in the *Zookeeper* version, participants order animal cards in a yellow and blue box, according to the color of a sticker applied on each card (OT); participants, in addition, place the elephant card in a white box set behind them (PM task) (Lavis and Mahy, 2021). Through this PM task, a relationship between PM and chronological age was identified as one of the indicators of the developmental process (Ford et al., 2012). The results of studies conducted by different authors have shown that older children tend to be more efficient, make fewer errors and have shorter reaction times compared to younger children. A significant difference was found between preschool-aged children and school-aged children (Kretschmer-Trendowicz et al., 2016; Kvavilashvili et al., 2001); older children showed better performance on PM tasks (Cejudo et al., 2022; Cejudo et al., 2019a; Mahy et al., 2016; Mahy and Moses, 2011; Zhang et al., 2019). It is important to note that there was no floor effect in the younger group, indicating that they were already able to perform PM tasks (Wang et al., 2008). The development of PM has been shown to depend on the maturation of executive functions, including updating, shifting, inhibition, monitoring, and working memory (Cejudo et al., 2022; Ford et al., 2012; Mahy et al., 2016; Mahy and Moses, 2011; Wang et al., 2008; Zhang et al., 2019). These functions were identified as crucial for the reallocation of attention and the adaptation of strategies to the difficulty of the task. In addition, retrospective memory has been observed to influence reaction times in PM tasks (Wang et al., 2008).

#### 3.2.2 Picture Classification Task

The *Picture Classification Task* is a paradigm proposed by Einstein and McDaniel (1990) used to evaluate event-based PM in the 3 to 16 years age range (Figure 3). The task is presented as a computer game, and participants classify objects on the computer screen according to categories organized in blocks. Each block starts with the presentation of the image and the name of the class to be referenced. Participants classify ideas by responding through specific keyboard keys if the object is part of the requested category. Children respond by pressing a different key (i.e., the spacebar) when a target image appears on the screen (PM task).

As for the previous paradigm, different and numerous versions have been proposed. In the *Karl and his dog Bubu* version, participants answer if an object belongs to the category indicated (OT). In this version, the classes are five, represented by rooms: kitchen, bathroom, children’s room, study room, and wardrobe. Children press a specific keyboard key (i.e., spacebar) when an image of a sandwich, candy, or umbrella appears on the screen (PM task). Other stimuli used in this version as PM cues are types of fruit (Cottini et al., 2018, 2019, 2021a,b). In the version adapted by Guajardo and Best (2000), the stimuli presented are colouring images divided into six blocks (OT). Children press a specific key on the keyboard (i.e., the spacebar) when duck or house images appear (PM task) (Guajardo and Best, 2000). In the version by Basso et al. (2023), participants looked at 266 black-and-white drawings of living and non-living objects, which were presented one after the other in 32 lists of different lengths. They were asked to memorize the last three images of each list without knowing the length of the list and to indicate whether a test image was among these last three images by pressing “yes” or “no” on the keyboard (OT). They were also asked to press the space bar when pictures of a pig, a belt or a pumpkin appeared (PM task) (Basso et al., 2023). In the *Shopping trip* version, participants see 34 photos of shops: 12 stores are the targets for shopping; 22 are photos of shops where participants should not stop (OT). Elmo, a character from Sesame Street, was featured in some images, and children captured the character with a specific keyboard key (PM task) (Walsh et al., 2014). In the *Arranging rooms for animals’* version, both time-based and event-based PM in children are evaluated. Participants should assign a corresponding colour (yellow, red, and blue) to the target category (dog, fish, and rooster) (OT). Children feed the rabbit with a carrot when the hourglass runs out (time-based PM task), and they provide the dog with a bone when the dog appears in the OT (event-based PM task) (Han et al., 2017). In the version used by Hartwig et al. (2021), participants see stills taken from the cartoons *Party Animals* and *Strictly no dancing* (OT). A bobble cap has been inserted in some stills. Children press a specific keyboard button each time they see a bobble cap (PM task) (Hartwig et al., 2021). The version adapted by Wang et al. (2024) is the only one that is exclusively time-based. Participants were shown 64 pictures of objects, animated or not. Participants had to determine whether the object in the picture shown was animated or inanimate by pressing the “F” or the “J” key on the computer (OT), respectively. Participants were asked to remember to press the “enter” key every 2 min (PM task) (Wang et al., 2024). In another version of this paradigm by Chen et al. (2017), participants observe the stimuli presented on the screen, 26 letters of the alphabet (OT). Children press a keyboard key when the letter “D” appears on the screen (PM task) (Chen et al., 2017). Another letter-based version was used by Kretschmer-Trendowicz et al. (2021), in which participants saw different sets of letter strings on the screen and had to indicate via the keyboard whether the presented strings were identical or not (OT). The children had to press a specific key on the keyboard when a sequence of letters “RLRLR” appeared on the screen (PM task) (Kretschmer-Trendowicz et al., 2021). Through this PM task, a relationship between PM and chronological age was identified as one of the indicators of the developmental process (Cottini et al., 2021a). The results of studies conducted by various authors have shown that older children tend to be more efficient and have shorter reaction times than younger children (Cottini et al., 2021a; Han et al., 2017). School-age children performed better than preschool children (Cottini et al., 2021b; Han et al., 2017; Hartwig et al., 2021). PM is subject to significant developmental changes during the primary school years (Chen et al., 2017). Results suggest that PM performance in children improves with age (Walsh et al., 2014); three-year-old children have at least a basic understanding of effective PM strategies, as even three-year-old children can use PM in a computer task (Guajardo and Best, 2000). Five-year-old children perform better than younger children on PM tasks at short and long intervals (Guajardo and Best, 2000). Twelve-year-old children were more efficient at performing the PM task compared to eight-year-olds (Cottini et al., 2021a). Using a reminder improved students’ PM accuracy (Chen et al., 2017). Executive functions, especially monitoring and working memory, were essential for successful PM in children (Cottini et al., 2018, 2019; Han et al., 2017; Hartwig et al., 2021). Attention is crucial in children’s PM tasks (Han et al., 2017). Metamemory was critical for PM development, as older children’s predictions and post-dictions were closer to actual PM performance than younger children’s; metamemory significantly improved children’s PM performance, primarily using predictions as strategies (Cottini et al., 2021a,b). Motivating children with rewards can improve their performance on PM tasks (Han et al., 2017). PM and RM performance of younger children were correlated (Han et al., 2017). PM and RM followed the same developmental trajectory. The contribution of retrospective memory promoted success in PM performance (Walsh et al., 2014).

#### 3.2.3 Cyber Cruiser

The *Cyber Cruiser* is a paradigm proposed by Kerns (2000) used to evaluate time-based PM in the 5 to 14 years age range (Figure 3). The task is presented as a computer game, and participants simulate driving a vehicle. They control the vehicle using a joystick or a computer keyboard. Participants get points by going fast on the track without hitting the other vehicles (OT) and monitoring the vehicle’s fuel (PM task). The vehicle’s power is refilled by pressing a specific button only when it becomes visible on display. In the *Cyber Cruiser* version, children drive a car while avoiding obstacles (OT) and refuel it when the fuel level in the tank is low (PM task) (Kerns, 2000; Kliegel et al., 2013; Mahy et al., 2015; Voigt et al., 2014; Voigt et al., 2015; Zuber et al., 2019). In the *Cyber Cruiser II* version, children drive a spaceship while avoiding obstacles (OT) and refuel the spacecraft when the fuel level in the tank is low (PM task) (Cheie et al., 2021). Studies show that the *Cyber Cruiser* task is suitable for a wide age range and makes cognitive demands that arouse children’s interest (Kerns, 2000; Kliegel et al., 2013). The *Cyber Cruiser* requires participants to observe the passage of time without explicit temporal cues, which encourages initiative and strategic monitoring (Cheie et al., 2021). School-aged children have shown better PM performance compared to preschool-aged children (Zuber et al., 2019). Therefore, older children show better PM performance compared to younger children (Cheie et al., 2021; Kerns, 2000; Kliegel et al., 2013; Mahy et al., 2015; Voigt et al., 2015). Possible ceiling effects were excluded in the older group (Kliegel et al., 2013). Executive functions such as updating, inhibition, shifting and monitoring improve with age and are crucial for PM performance (Cheie et al., 2021; Kerns, 2000; Kliegel et al., 2013; Mahy et al., 2015; Voigt et al., 2015; Zuber et al., 2019). Children performed worse under conditions of divided attention than under conditions of sustained attention (Mahy et al., 2015). Retrospective memory has been identified as a valuable component in task performance (Kliegel et al., 2013).

#### 3.2.4 Virtual Week

The *Virtual Week* is a paradigm adapted from Rendell and Craik (2000) used to assess both time-and event-based PM in the 7 to 12 years age range (Figure 3). The task is presented as a computer board game and is a simulation of everyday situations in a virtual week. During the game, participants roll a die to move a token on a board and make choices between various alternatives offered by the game, such as choosing their preferred breakfast (OT). Children complete tasks that are typical of their daily lives, which are presented on the board and on activity cards. These tasks can be time-based, such as using an inhaler for asthma at 11:00 and 21:00 (time-based PM task). They can also be event-based, such as taking antibiotics at breakfast and dinner (event-based PM task). Participants must complete eight PM tasks in each ‘virtual day’ of the game. Four of these tasks are regular, consisting of two time-based PM tasks and two event-based PM tasks that are common in the lives of children. The other four tasks are considered irregular, consisting of two time-based PM tasks and two event-based PM tasks that are less frequent in the life of children (Terrett et al., 2019; Yang et al., 2011). Between the ages of 7 and 11, PM performance improved linearly and stabilized at age 12, with occasional errors. The first major improvement occurred between the ages of 7 and 8, with an increase in the frequency of recall of PM tasks and fewer errors of omission (Yang et al., 2011). Between the ages of 8 and 10, development was slower but steady, with a decrease in omission errors and a significant reduction in repetition errors, indicating a qualitative improvement. At age 10 to 11 years, there was a further significant improvement with an increase in the number of remembered PM tasks, and the level of PM performance remained stable until the age of 12 (Yang et al., 2011). The concentration and reading demands of the Virtual Week game make it unsuitable for children under the age of 8, and reliability has only been established for children aged 8 to 12 (Terrett et al., 2019). Retrospective memory supported PM performance by enhancing the inclusion of details of the PM task (Terrett et al., 2019). Children’s accuracy in PM tasks improved with age. Executive functions, particularly working memory and inhibition, were correlated with PM (Yang et al., 2011).

#### 3.2.5 Fishing Game

The *Fishing Game* is a paradigm proposed by Yang et al. (2011) to assess both time-and event-based PM in the 6 to 12 years age range (Figure 3). The task is presented as a computer game and participants catch as many fish as possible to earn points (OT). The children were exposed to two different game conditions. In the first condition, children stop fishing when they encounter a fish with certain characteristics to feed the cat next to the game avatar (event-based PM task). In the second condition, children stop the current activity when the digital clock in the upper right corner of the screen reaches the full minute to feed the cat next to the game avatar (time-based PM task) (Cheie et al., 2021; Yang et al., 2023; Yang et al., 2011). Using this PM task, it was found that performance increased with age (Cheie et al., 2021; Yang et al., 2011). Children aged 6 to 10 years had to perform the PM action when the digital clock showed 1:00, 2:00 and 3:00. These processes May be related to children’s learning of time and numerical skills (Cheie et al., 2021; Yang et al., 2011). The *Fishing Game* might be a better choice for cross-cultural studies as it requires less linguistic effort and only simple mouse operations (Yang et al., 2011). Executive functions – such as updating, inhibition, shifting, monitoring and working memory – improved performance in PM (Cheie et al., 2021; Yang et al., 2011).

#### 3.2.6 Ask for stickers

The *Ask for stickers* is a paradigm used to evaluate event-based PM in the 2 to 15 years age range (Figure 3). The task is presented as a game. Participants play with an experimenter performing different tasks or games (OT) and must remember to pick up a gift (stickers or snacks) when the experimental session ends (PM task) (Causey and Bjorklund, 2014; Guajardo and Best, 2000; Hashimoto et al., 2022; Kelly et al., 2023; Ślusarczyk et al., 2018; Ślusarczyk and Niedźwieńska, 2013; Walsh et al., 2014). Using this PM task, it was found that children’s PM performance improved with age (Walsh et al., 2014). Older children showed better PM performance compared to younger children (Guajardo and Best, 2000). PM performance improved systematically during the preschool years (Ślusarczyk and Niedźwieńska, 2013). Preschool-aged children can fulfil a delayed intention if they are motivated to do so (Causey and Bjorklund, 2014). Two-year-old children can perform PM tasks to a certain extent if the motivation to complete the task is sufficiently high (Ślusarczyk and Niedźwieńska, 2013). Two-year-old children are not able to benefit from longer retention intervals (Ślusarczyk et al., 2018). The incentive for children to retain the object to be memorized could explain the performance of three-year-olds (Guajardo and Best, 2000). The children remembered asking for the sticker more often than asking to close the door or asking for the pencil. This result suggests that interest in obtaining the sticker facilitated remembering this task (Guajardo and Best, 2000). Preschool children showed good PM when the task was important to them (Causey and Bjorklund, 2014). Children needed multiple reminders to recall the intention (Kelly et al., 2023). Executive functions, especially working memory and inhibitory control, were essential for the development of PM (Causey and Bjorklund, 2014; Guajardo and Best, 2000; Ślusarczyk and Niedźwieńska, 2013). Differences in the development of executive functions, especially working memory, could also explain differences in PM performance in preschool-aged children (Guajardo and Best, 2000). High motivation was necessary for two-year-olds to perform well and remained an important factor that increased performance throughout the preschool years (Causey and Bjorklund, 2014; Guajardo and Best, 2000; Ślusarczyk and Niedźwieńska, 2013). PM and retrospective memory performance were correlated in younger children (Guajardo and Best, 2000). The contribution of retrospective memory facilitated success in PM performance (Walsh et al., 2014).

#### 3.2.7 Sightseeing tour

The *Sightseeing tour* is a paradigm used to evaluate both time-based and event-based PM in the 10 to 12 years age range (Figure 3). The task is presented as a game. Children participate in a *Sightseeing tour* structured in four attractions (OT) and remember to carry out activities required by the experimenter (PM task). The participants are on a bridge during the first attraction and must throw balls in a bucket. Participants stack small wooden sticks at the second attraction to build a tower as high as possible. During the third attraction, participants copy a painting. Finally, the fourth stop is dedicated to solving enigmas with matches (OT). Children receive six instructions for prospective activities: three *social* PM tasks with experimenter interaction (S) and three *neutral* PM tasks without interaction (N). S1: give the experimenter a handkerchief when sneezing; S2: fill one glass of water as soon as the experimenter empties it; S3: provide the experimenter with a pen at the end of the tour. N1: put a ticket in a box at the end of the tour; N2: pin an image on a bulletin board when the experimenter places a photo on a red chair; N3: wear a jacket when the experimenter opens the window (PM task) (Zhang et al., 2019). Using this PM task, it was found that PM performance improved in childhood (Kretschmer-Trendowicz et al., 2019). Children May not yet have acquired the competence to spontaneously apply advanced strategies, whereas adolescents, due to their greater knowledge of strategies, can use them to ensure good performance when given additional time (Kretschmer-Trendowicz et al., 2019). Research has shown no significant differences between neutral and socially delayed intentions in PM tasks (Kretschmer-Trendowicz et al., 2019). Retrospective memory, especially by encoding future episodic thoughts as a strategy, contributed to the improvement of PM (Kretschmer-Trendowicz et al., 2019).

#### 3.2.8 MISTY

The *MISTY* is a paradigm adapted by Raskin et al. (2010) used to assess both time-based and event-based PM in the 4 to 15 years age range (Figure 3). The task is presented as a paper-and-pencil test. The MISTY is structured similarly to the MIST (Raskin et al., 2010), a test employed in neuropsychology to assess prospective memory in adults. In the MISTY, participants solve a crossword puzzle (OT) while performing eight PM tasks. Half of the tasks aim to assess time-based PM (e.g., “in two minutes, at 3:05, tell me to do my homework”), while the other half aim to assess event-based PM (e.g., “when I hand you a blue pen, draw a house”) (PM task) (Mills et al., 2021). Additionally, children must answer eight multiple-choice questions presented by the experimenter (e.g., “When I gave you a blue marker, were you supposed to…? 1. Draw a house? 2. Draw a picture of your family? 3. Draw a cat?”), which are administered to test recognition memory (Mills et al., 2021). Using this PM task, it was found that PM performance increased significantly with age (Mills et al., 2021). Comparisons between age groups 4–6, 7–8, 9–10, 11–12 and 13–15 years showed significant differences in overall PM performance, indicating consistent improvements with age (Mills et al., 2021). These improvements are related to the development of retrospective memory, working memory and executive functions (Mills et al., 2021). The good reliability between the items and subscales of the MISTY indicates that they consistently measure the same construct.

#### 3.2.9 Mouse task

The *Mouse task* is a paradigm adapted from Lejeune et al. (2013) used to evaluate time-based PM in the 4 to 9 years age range (Figure 3). The task is presented as a computer game, and participants use the inverted mouse to interact with the stimuli on the screen. In the “easy” version, participants capture the image of a cartoon character appearing on the screen and place it in a basket at the bottom of the screen. In the “hard” version, participants outline a triangular shape and capture several toys that appear on the screen (OT). Children must remember to press a specific key on the computer keyboard (i.e., spacebar) within 30 s whenever cartoon characters reach a red area on the screen (PM task) (Geurten et al., 2016). Executive functions, especially monitoring, had a positive impact on children’s PM performance. Knowledge of working memory strategies can be used to improve PM performance in childhood (Geurten et al., 2016).

#### 3.2.10 The puzzle/reading task/find the differences/math problems task

The *Puzzle/Math problems/Reading task/Find the differences* is a paradigm used to evaluate event-based PM in the 6 to 13 years age range (Figure 3). The task is presented as a game, and participants perform four tasks in a constant order: a puzzle, reading, find differences, and math problems (OT). The experimenter asks the children to remember each task’s stimulus (PM task).

In the puzzle task, participants complete a puzzle (OT); at the end of the task, children must remember to put all the puzzle pieces in the box except for two unused pieces (PM task). In the reading task, participants read sentences from a notebook and emphasize words that refer to animals (OT); they must remember to circle the words referring to numbers (PM task). In the find the differences task, participants find differences between two images (OT); they must indicate with an arrow the most challenging difference to find (PM task). In the math problems task, participants solve math problems (OT); they must circle number 3 (PM task) (Cejudo et al., 2019b). In Shum et al. (2008), participants read a story (OT). The 8–9 year olds read a story entitled “The Twig Fence” and had to replace the character’s name “Henry” with “Tom” (PM task), while the 12–13 year olds read “The Fire” and had to replace the term “Lower Palmer” with “Upper Palmer” (PM task) (Shum et al., 2008). This PM task showed that older children achieved better results than younger children. The reaction times indicated that maintaining intention was a greater challenge for younger children. Accuracy in task performance also showed a similar pattern, with differences between younger and older children depending on the focality of the cue (Cejudo et al., 2019b). From a young age, children can use preparatory attentional processes and monitoring strategies for PM cues that influence their performance on the OT. These improvements are likely related to the development of executive functions in childhood. Moreover, motivation played a crucial role in PM performance during their development (Cejudo et al., 2019b).

#### 3.2.11 Reminding task

The *Reminding task* is a paradigm used to evaluate both time-based and event-based PM in the 2 to 4 years age range (Figure 3). The task is presented as a verbal request. Caregivers provide indications of activities to remember, only once. Every day the caregiver gives participants 10 min to remember to do the activity without help. Participants must remember to carry out prospective activities in everyday life (OT). Children remember to perform caregivers’ requests at the appropriate times (i.e., 3:30 pm; time-based PM task) or at a suitable event (i.e., “when Dad comes home”; event-based PM task) (Somerville et al., 1983). The success in retrieving PM performance was evident even in younger children. Children aged 2, 3, and 4 showed remarkable competence in deliberately recalling the tasks presented to them (Somerville et al., 1983). On average, they recalled high-interest tasks with a short delay without prompting 73% of the time, with 2-year-olds recalling without prompting 80% of the time. These results suggested that the children were able to spontaneously recall high-interest tasks. Overall, performance on high-interest tasks was significantly higher than on low-interest tasks. In addition, motivation and high-interest tasks promoted success in retrieving PM tasks, even in younger children (Somerville et al., 1983).

## 4 Discussion

PM is an essential skill for the lifelong autonomy of individuals. Specifically, during development, it is critical for children’s daily functioning such as school performance, social relationships, and their ability to become independent from caregivers (Mahy et al., 2014a). For relevance played by PM in development, it seemed crucial to summarize the evidence in literature. The main aim of the present systematic review was to identify and describe the tasks used to evaluate PM in childhood and adolescents, potentially offering indications to researchers regarding the optimal tasks to measure PM in different ages (see Figure 3). Moreover, it aimed to provide a general overview of the development of PM in children and adolescents as well as to increase the knowledge on the cognitive functions involved in PM, through an overview of the specific assessments carried out in the primary studies included.

### 4.1 Prospective memory tasks to evaluate PM in developmental age

The main objective of the present review was to identify and describe the tasks used to evaluate PM in children and adolescents. The forty-nine studies included in the present review used many different tasks that can be traced back to eleven different main paradigms to evaluate PM (see Figure 3): an ongoing task (OT) and a prospective memory activity (PM task) characterized the tasks used. In most studies, the activities were presented as a game to keep children’s attention and motivation high. Importantly, within these eleven paradigms identified, some PM tasks varied slightly from the reference task. Indeed, only some features of the game presented to the participants differed, such as the cue stimulus of PM. For example, Cheie et al. (2021) adapted *Cyber Cruiser* (Kerns, 2000), in which participants had to drive a car (OT) and refuel the vehicle (PM task), by substituting a spaceship instead of the car to create their *Cyber Cruiser II* version. So, with the aim to map and synthesize the different tasks used, all versions of the “same” task have been merged in a single category (paradigm). Specifically, this merger was based on two criteria: first, if the task was adapted from a task previously used in literature and the authors reported the original source [e.g., the *Dresden Cruiser* used by Voigt et al. (2015), was an adaptation of the *Cyber Cruiser* originally developed by Kerns (2000)]; second, if tasks had the same PM activity, even though the OT was different [e.g., there are several versions of the *Picture Classification Task*, originally developed by Einstein and McDaniel (1990). The initial version involved participants assisting the protagonists of the game, Karl and his dog Bubu, in packing their backpacks for a school trip (OT), remembering to fill the backpack with the items listed on a checklist (PM task). The *Shopping Trip*, used by Walsh et al. (2014), was an adaptation of the *Picture Classification Task*, in which participants were asked to shop at certain shops instead of others (OT), remembering to purchase the items listed on a checklist (PM task). Each version had different backgrounds (OT), but the basic PM task remained identical: children had to respond by pressing a different key (e.g., the space bar) when a target image appeared on the screen (PM task)].

Importantly, some of the tasks used in the primary studies only evaluated the event-based PM (e.g., *Card Sorting game, Picture Classification task, Ask for stickers, Sightseeing tour, Puzzle/Math problems/Reading task/Find the differences task*), others only evaluated the time-based PM (e.g., *Cyber Cruiser, Mouse task*), and still others evaluated both the time-based and event-based PM (e.g., *Virtual Week, Fishing Game, MISTY, Remembering Task*) offering a more accurate assessment (see Table 1).

Based on the literature reviewed, we now tried to highlight the strengths and weaknesses of the different paradigms as well as to offer indications to researchers in selecting optimal tasks for measuring PM across different age groups (Figure 3).

The *Card Sorting Game* is a paradigm that adequately grasps the essential features of PM and allows us to obtain quantitative PM measures. In addition, this task has sufficient sensitivity to detect age-related changes in children (Mahy and Moses, 2015) and it appears appropriate to evaluate PM starting from 2 years of age.

The *Picture classification task* is a laboratory task, both motivating and ecological, which measures children’s ability to remember to act in the future. Still, it May evaluate planning less than a more naturalistic task involving a longer delay, more distractions, and competing responses (Chen et al., 2017; Cottini et al., 2018; Cottini et al., 2021b; Guajardo and Best, 2000; Han et al., 2017). It is important to underline that, based on the literature reviewed, this task seems to be a useful tool for assessing PM in a wide age range (from 2 to 16 years).

The *Cyber Cruiser* controls the participant’s behavior from when the intention is formed to its execution. This paradigm is appropriate to assess PM in a wide range of ages, from childhood to adolescence (Kerns, 2000), as participants between 5 and 14 years old did not show floor or ceiling effects in this task (Mahy et al., 2015).

The *Virtual Week* was designed to mimic an environment with continuous stimuli of daily life rather than a consecutive presentation of artificial objects (triggers) typically used in laboratory activities. Most of the *Virtual Week* PM activities closely resembled the children’s daily activities and were realistic in reminding children of real-life consequences. This tool requires a verbal reminder and lets you grasp qualitative and quantitative aspects. Furthermore, this task allows us to identify the relationship between different types of PM activities, both regular and irregular. This paradigm was also developed to increase the validity of the evaluation and to provide accuracy and error measurements for all PM types (Yang et al., 2011). However, the task requires concentration and reading, making it unsuitable for children under 8. The reliability of this paradigm has only been established for children aged 8 to 12 years (Henry et al., 2014; Terrett et al., 2019).

The *Fishing Game* is a typing task used in a laboratory to assess PM development in children from 6 to 12 years (Yang et al., 2011). The task is characterized by the presentation of consecutive artificial objects and it reflects the quantitative aspect of PM.

The *Ask for Stickers* is a naturalistic task used to evaluate PM and it seems to be appropriate from preschool age to adolescence. As opposed to computer tasks—which measure children’s ability to remember to act in the future, but May not consider any delay in intention, distractions, and competing responses—naturalistic tasks mirror everyday activities. The stickers, or snacks, used as rewards, were attractive to younger children, leading them to a more excellent PM performance on that task. Indeed, incentives could be effective when tasks are challenging (Guajardo and Best, 2000). Therefore, due to the wide age range of application, ecological validity and ease of administration, the *Ask for Stickers* can be considered a very useful activity to assess PM in many different contexts.

The *Sightseeing tour* is a complex task, with real, neutral, and social PM tasks, in which the social aspect was manipulated implicitly. Specifically, the tasks required individuals to do something for a third person (for example, give the experimenter a handkerchief when sneezing). However, children were not explicitly aware of the importance of these tasks (Kretschmer-Trendowicz et al., 2019). Probably because of its complexity, this test was used with participants aged 10 to 12 years.

The MISTY is a paper and pencil evaluation tool, and it is the only task not presented as a game. Participants must solve a crossword puzzle (OT) while performing four time-based and four event-based PM tasks. The psychometric properties of this test are adequate to promote the use of MISTY as a clinical measure; in fact, it represents a potential innovative opportunity for the clinical evaluation of PM in children and adolescents (Mills et al., 2021). Finally, the MISTY appears to be a tool suitable for a wide age range; in fact, it was used from childhood to adolescence (4–15 years).

In the *Mouse task*, children were instructed to use the computer mouse in reverse mode (i.e., the mouse is positioned upside down) to “capture” the stimulus and put it in a basket at the bottom of the screen as quickly as possible. The OT provided an easy version and a difficult one based on age (Geurten et al., 2016). This test has proven to be appropriate for assessing PM in the age range in which it has been used so far (4–9 years).

In *Puzzle/Math problems/Reading task/Find the differences task* children were asked to remember four tasks in natural school contexts, where children move more freely and information is more scattered, compared to computer tasks that are more absorbing (i.e., more focalizing) than realistic tasks (Cejudo et al., 2019b). Probably due to their features, these tasks have not been used before the age of 6.

In the *Reminding task*, caretakers had to use a written and standardized diary to record whether the child remembered unaided the activities to be performed. Still, it was impossible to formally verify the observational reliability of the caretakers (Somerville et al., 1983). For its easy structure, this task seems to be appropriate to obtain an early PM assessment (2–4 years).

### 4.2 Development of PM

A further proposal of the present systematic review was to provide a general overview of the development of PM. From what emerges, the heterogeneity of tools used May have contributed to the difficulties in tracing the development of this cognitive function. Indeed, a standardized approach could provide the necessary control to eliminate the influence of confounding variables (Kvavilashvili et al., 2001). Researchers found a higher PM performance in older children than younger children (Cejudo et al., 2019a; Cheie et al., 2021; Hartwig et al., 2021; Voigt et al., 2015), and no differences were found between males and females (Mills et al., 2021; Yang et al., 2011). Moreover, the studies included seem to agree on the fact that older children were more accurate and had shorter reaction times when performing the OT while trying to remember the intention of the PM goals than younger children (Cejudo et al., 2019a; Cottini et al., 2021b; Kretschmer-Trendowicz et al., 2016; Kvavilashvili et al., 2001; Zimmermann and Meier, 2006). These results could be due to several factors. First, the retrospective failure rate in younger children was higher. Second, the OT task, identical for different age groups, most likely made the task easier for older children. In addition, children’s PM performance improves with age and varies with OT cognitive demands (Geurten et al., 2016; Kretschmer et al., 2014; Voigt et al., 2015; Ward et al., 2007; Yang et al., 2011; Zimmermann and Meier, 2006), so older children May have been monitoring the PM cue, while younger children failed to do so (Hartwig et al., 2021). The superior performance of older children probably relied on more effective proactive control strategies rather than reactive control strategies (Mahy et al., 2015). Early childhood is a crucial period for the development of an individual’s PM capacity (Guajardo and Best, 2000; Han et al., 2017; Kliegel and Jäger, 2007); the need to remember to carry out a planned action in the future was, indeed, linked to the fact that PM tasks were incorporated within an interpersonal network, and their success was socially rewarded (Ślusarczyk et al., 2018). According to several authors, one might expect PM to manifest itself early in development (Meacham and Colombo, 1980; Ślusarczyk et al., 2018; Winograd, 1988). The first signs of PM ability are already seen in two-year-olds (Guajardo and Best, 2000; Kliegel and Jäger, 2007; Rendell et al., 2009; Ślusarczyk et al., 2018; Ślusarczyk and Niedźwieńska, 2013; Somerville et al., 1983; Wang et al., 2008; Yang et al., 2011; Zimmermann and Meier, 2006). The PM of two-year-old children was substantial when they were highly motivated to perform the PM task (Ślusarczyk et al., 2018; Ślusarczyk and Niedźwieńska, 2013), although children of this age May not be able to update their intentions efficiently during a retention interval due to limited executive functions and poor monitoring capacity (Ślusarczyk et al., 2018). Children under the age of three could use PM in both naturalistic and laboratory tasks (Guajardo and Best, 2000). Preschoolers could perform not only familiar PM tasks, such as reminding the experimenter or caregiver to provide him with something he liked, but they could perform these tasks even when they faced a new situation (unfamiliar PM tasks), such as pressing a key when an image appeared on the computer screen (Guajardo and Best, 2000). When performing PM activities, three-year-old children demonstrated a rudimentary understanding of effective strategies; this suggested that they had at least an elementary understanding of these PM strategies (Guajardo and Best, 2000). The difference in children’s PM performance between three and four was not significant (Han et al., 2017). In the fourth year of life, there was a development in cognitive abilities that supported the propensity of children to perform tasks that did not involve an immediate or salient profit (Causey and Bjorklund, 2014). Four-year-old children were intermediate between younger and older children (Walsh et al., 2014). The PM of four-year-olds was worse than the PM of five-year-olds, who outperformed three-and four-year-olds in PM tasks (Han et al., 2017; Mahy et al., 2014b; Mahy and Moses, 2015; Zhang et al., 2017). The age of five is crucial in developing PM skills (Mahy et al., 2014b). These children did not have a floor effect on PM tasks. Five-year-old children performed better in PM tasks because they had a more developed understanding of strategies than three-year-old children (Guajardo and Best, 2000). This interpretation is consistent with data showing that correlations between retrospective memory strategies and performance increased with age (Weinert and Schneider, 1995). Indeed, a greater understanding of strategies used by older children could be a precursor to their further development of potential memory capacities (Guajardo and Best, 2000). At seven years of age, children were increasingly able to use active strategies (Lavis and Mahy, 2021; Lehmann and Hasselhorn, 2007; Schneider and Pressley, 2013). Indeed, the first significant improvement occurred between seven and eight years of age, showing a high frequency of remembering PM activities and fewer forgetfulness errors (Yang et al., 2011). From age eight to ten, growth was slower but accompanied by a steady decrease in forgetfulness errors and a significant reduction in repeat errors, suggesting qualitative growth (Yang et al., 2011). Nine-and ten-year-old children remembered executing delayed intentions better than six-and seven-year-olds (Kliegel et al., 2013). From the age of ten to eleven, there was another significant improvement, with an increasing number of PM tasks maintained until the age of twelve (Yang et al., 2011). The development of PM continues in adolescence. In adolescents, success in PM improves considerably (Hashimoto et al., 2022). Eleven-year-old children have shown better performance on PM tasks compared to younger children (Shum et al., 2008), including time-based prospective memory (TBPM), suggesting that TBPM ability continues to develop during the school years (Yang et al., 2023). Adolescents aged 11 and 15 years show significantly better performance than younger school-age children (Hashimoto et al., 2022; Kretschmer-Trendowicz et al., 2021). Beyond that age, adolescents behaved like adults in PM performance (Cejudo et al., 2019a; Kerns, 2000). The increase of reaction speed with age and the improved management of cognitive resources makes PM tasks more efficient and accurate (Kretschmer-Trendowicz et al., 2021). This improvement is linked to the development of executive functions such as future thinking, metacognition and the ability to switch between different tasks. These skills enable adolescents to better manage their future intentions while continuing to complete ongoing tasks (Hashimoto et al., 2022).

### 4.3 The cognitive process involved in the development of PM

The ultimate proposal of this systematic review was to increase the knowledge on the cognitive functions involved in PM, through an overview of the specific assessments carried out in the primary studies included, which have shown mixed results. Despite this conflicting and multifaceted literature, various studies have suggested the involvement of different cognitive functions; among these, the most studied ones were retrospective memory, metamemory, executive functions, motivation, and attention.

#### 4.3.1 Retrospective memory

Starting from retrospective memory, there were inconsistencies among different studies (Wang et al., 2008). In the study by Kvavilashvili et al. (2001) regression analyses did not show a relationship between children’s PM and retrospective memory scores, as retrospective memory did not significantly affect PM performance (Wang et al., 2008). Some studies showed that the two forms of memory were not correlated (Brandimonte and Passolunghi, 1994; Einstein and McDaniel, 1990; Guajardo and Best, 2000; Kidder et al., 1997), while other studies showed that PM and retrospective memory were closely related during the early stages of cognitive development (Ślusarczyk et al., 2018). Mahy et al. (2014b) suggested that retrospective memory involvement was necessary to recall predicted intention (Zhang et al., 2017). Indeed, this could have repercussions for preschoolers who did not remember the PM task instructions at the end of the procedure (Kliegel and Jäger, 2007; Zhang et al., 2017). Retrospective memory was involved in the development of PM, as children, thanks to this cognitive function, remembered what had to be done (Guajardo and Best, 2000). So, retrospective memory supported PM performance, improving the ability to code and maintain PM task requirements. The formation of more detailed intentions during the coding phase of a planned intention could increase the probability that the action would be performed later (Chasteen et al., 2001; Kliegel et al., 2000; Zhang et al., 2017). Individuals, therefore, needed to retrospectively recall the associations between cues and intentions to perform, thus initiating the PM process (Mills et al., 2021).

#### 4.3.2 Metamemory

Metamemory also seemed necessary for the proper functioning of PM at developmental age. When a task had to be remembered in the future, it helped to construct, imagine, or remember it in advance; all these activities are based on metamemory (Causey and Bjorklund, 2014; Cejudo et al., 2019a; Geurten et al., 2016). Although children generally overestimate their specific performance (Cottini et al., 2019), the ability to form a flexible mental representation of an expected task and the environmental conditions that allow for its execution, appear to facilitate PM (Causey and Bjorklund, 2014). Simulating or imagining future events has been a successful PM strategy for school-age children (Causey and Bjorklund, 2014; Cottini et al., 2018). Kvavilashvili and Ford (2014) believed that PM performance was more accurate in children who better predicted their performance because they chose predictions as the most appropriate strategy to tackle the PM task (Cejudo et al., 2019a). Indeed, the relationship between performance predictions and actual performance was significant (Kvavilashvili and Ford, 2014). Performance predictions could be used, at school age, to facilitate the success of PM goals by facilitating the execution of the planned intention.

#### 4.3.3 Executive functions

The studies included in this systematic review also showed an essential role of EF as possible mechanisms underlying the observed improvements in PM development (Cottini et al., 2019; Geurten et al., 2016; Hartwig et al., 2021; Shum et al., 2008; Voigt et al., 2015; Walsh et al., 2014). PM development in childhood depends mainly on the development of executive functioning (Cheie et al., 2021; Zuber et al., 2019). Rather than age itself, EF led to improved PM performance during childhood (Atance and Jackson, 2009; Ford et al., 2012; Kerns, 2000; Wang et al., 2008; Zuber et al., 2019). Indeed, the immaturity of these functions and underlying brain structures likely limited PM in young children (Walsh et al., 2014), especially under conditions of high cognitive demand during OT (Mahy and Moses, 2015; Shum et al., 2008; Ward et al., 2007). Different EF—working memory, inhibition, set switching, planning, and monitoring—played more prominent roles than others at certain PM stages (Mahy et al., 2014a), resulting in initial encoding, retention, and retrieval of intentions (Chen et al., 2017). Inhibitory control predicted performance accuracy (Cottini et al., 2018; Mahy and Moses, 2015) and was significantly correlated with PM of children and adolescents (Zuber et al., 2019). Inhibitory mechanisms, in developmental age, were necessary to interrupt the OT and allow for other intended actions, ignoring irrelevant information (Cheie et al., 2021; Kerns, 2000; Kliegel et al., 2013; Voigt et al., 2015; Zuber et al., 2019). The distribution of cognitive resources and children’s success in PM tasks also depended on refresher resources, mainly involved in PM action planning, regularly maintaining and reactivating OT and PM activity instructions (Mahy et al., 2014a; Zuber et al., 2019; Cheie et al., 2021). The dual-task situation, OT and PM, overloads the cognitive abilities of young children (Voigt et al., 2015), so strategic monitoring was a crucial process to ensure the success of a PM activity (Voigt et al., 2015; Zuber et al., 2019) There was a positive relationship between the strategic time monitoring score and the performance of PM even when their resources were involved in a cognitively challenging OT (Costa et al., 2010; Geurten et al., 2016; Mäntylä et al., 2007; Voigt et al., 2015; Zinke et al., 2010). Several studies have found a relationship between working memory capacity and PM (Ford et al., 2012; Hartwig et al., 2021; Mahy and Moses, 2011; Yang et al., 2011). Working memory is another critical factor in prospective memory’s accuracy and success (Cottini et al., 2018). Increasing this ability in older children supported the effectiveness of keeping the intention active (Hartwig et al., 2021), keeping the information in mind even during a delay (Kerns, 2000). Finally, displacement skills can support information monitoring, ensuring more flexible shifts between OT and PM tasks (Kliegel et al., 2013; Voigt et al., 2015).

#### 4.3.4 Motivation

Motivation had a powerful impact on PM in childhood, both for preschool and schoolchildren (Ślusarczyk et al., 2018). Studies that considered the relationship between PM and motivation showed that increasing motivation, both verbally and with material rewards, significantly improved PM performance (Han et al., 2017; Ślusarczyk et al., 2018). Children were susceptible to incentive manipulation (Causey and Bjorklund, 2014; Guajardo and Best, 2000; Ślusarczyk et al., 2018); in fact, the results showed more success in performing a PM task when it was personally rewarding to do so (Causey and Bjorklund, 2014). In addition, young children, compared to older children, had more limited cognitive resources, so motivation could help allocate their cognitive resources to the salient aspects of a task (Zhang et al., 2017).

#### 4.3.5 Attention

As for attention, the results indicated a significant impact of age and attention on PM performance (Cejudo et al., 2019a; Cottini et al., 2021b; Cottini and Meier, 2020; Mahy et al., 2015; Smith et al., 2010). Typically, school-age children could actively adjust the allocation of their attention resources to different tasks depending on their level of motivation (Han et al., 2017). The allocation of attentive resources in children when completing a PM task was an organic combination of both “top-down” and “bottom-up” processes (Han et al., 2017). Older children can shift their focus more flexibly between the OT (attentional focus) and the future goal. Younger children seemed to have difficulty with these changes, which resulted in the detection of fewer PM signals (Mahy et al., 2015).

## 5 Conclusion

In conclusion, this systematic review represents a fundamental starting point for the synthesis of instruments used in the assessment of PM in children and adolescents. It clearly highlights the urgent need to adopt standardized instruments to conduct accurate assessments of PM.

The diversity of paradigms and methodologies, as well as the lack of their proven psychometric properties and diagnostic accuracy, could be a limitation in three crucial aspects in the study of PM in children and adolescents. Firstly, for the assessment of PM, the lack of a single instrument emerges as a limitation, both for the types of PM analysed and the methodologies used. Secondly, the diversity of the instruments adopted could be a possible cause of the difficulties encountered in defining a precise development curve of this cognitive function. Thirdly, the multiplicity of instruments used to assess both PM and different cognitive functions has made it difficult to achieve a clear understanding of the role played by each cognitive function in the development of PM.

A standardized approach, as proposed by Kvavilashvili et al. (2001), with clear psychometric properties and diagnostic accuracy, could offer the necessary control to mitigate the impact of confounding variables that have led to gaps in the current state of the art. The present review sought to bridge these gaps in the literature. The findings reveal a J-shaped curve in PM development, as the first signs of PM are seen in 2-year-olds, while adolescents perform similarly to adults in PM tasks. Moreover, the present review delineated the cognitive processes—including executive functions, retrospective memory, metamemory, attention, and motivation—crucial to the development of PM in childhood and adolescents. Nevertheless, it is crucial to underscore how the diversity in the inclusion and exclusion criteria employed across various studies combined to the heterogeneity of instruments used to assess not only PM, but also other cognitive functions, presented a notable challenge in generalizing the conclusions drawn in this systematic review.

To summarize, the outlined recommendations provide a clear framework for future research on prospective memory in children and adolescents. Standardization of assessment instruments is essential to ensure the reproducibility, consistency and comparability of the data collected and to effectively address the current gaps in the literature. The importance of conducting longitudinal studies cannot be overstated, as they allow the observation of PM development over time and the identification of factors that influence this dynamic process. It is equally important to investigate the role of different cognitive functions in PM development, considering age-related differences. This multidimensional approach will enable a deeper understanding of PM development in different contexts. These findings will not only enrich the scientific literature and significantly improve our understanding of PM dynamics in children and adolescents, but could also pave the way for targeted interventions to promote PM during critical periods of growth and development.

## Data Availability

The original contributions presented in the study are included in the article/supplementary material, further inquiries can be directed to the corresponding authors.

## References

[ref1] Atance C. M. Jackson L. K. (2009). The development and coherence of future-oriented behaviors during the preschool years. J. Exp. Child Psychol. 102, 379–391. doi: 10.1016/j.jecp.2009.01.001, PMID: 19232416

[ref2] Basso D. Corradini G. Cottini M. (2023). “Teacher, forgive me, I forgot to do it!” the impact of children's prospective memory on teachers' evaluation of academic performance. Br. J. Educ. Psychol. 93, 17–32. doi: 10.1111/bjep.12537, PMID: 35934815 PMC10087291

[ref3] Bisiacchi P. S. Tarantino V. Ciccola A. (2008). Aging and prospective memory: the role of working memory and monitoring processes. Aging Clin. Exp. Res. 20, 569–577. doi: 10.1007/BF03324886, PMID: 19179842

[ref4] Brandimonte M. A. Einstein G. O. McDaniel M. A. (eds.) (2014). Prospective memory: theory and applications. *1st Edn*. New York, NY: Psychology Press.

[ref5] Brandimonte M. A. Ferrante D. Bianco C. Villani M. G. (2010). Memory for pro-social intentions: when competing motives collide. Cognition 114, 436–441. doi: 10.1016/j.cognition.2009.10.011 19913218

[ref6] Brandimonte M. A. Passolunghi M. C. (1994). The effect of cue-familiarity, cue-distinctiveness, and retention interval on prospective remembering. Q. J. Exp. Psychol. 47, 565–587. doi: 10.1080/14640749408401128, PMID: 7938668

[ref7] Burgess N. Becker S. King J. A. O’Keefe J. (2001). Memory for events and their spatial context: models and experiments. Philos. Trans. R. Soc. Lond. B Biol. Sci. 356, 1493–1503. doi: 10.1098/rstb.2001.0948 11571039 PMC1088531

[ref8] Burgess P. W. Scott S. K. Frith C. D. (2003). The role of the rostral frontal cortex (area 10) in prospective memory: a lateral versus medial dissociation. Neuropsychologia 41, 906–918. doi: 10.1016/S0028-3932(02)00327-5, PMID: 12667527

[ref9] Carlson S. M. Davis A. C. Leach J. G. (2005). Less is more: executive function and symbolic representation in preschool children. Psychol. Sci. 16, 609–616. doi: 10.1111/j.1467-9280.2005.01583.x 16102063

[ref10] Causey K. B. Bjorklund D. F. (2014). Prospective memory in preschool children: influences of agency, incentive, and underlying cognitive mechanisms. J. Exp. Child Psychol. 127, 36–51. doi: 10.1016/j.jecp.2014.01.020 24813540

[ref11] Cejudo A. B. Gómez-Ariza C. J. Bajo M. T. (2019a). The cost of prospective memory in children: the role of cue focality. Front. Psychol. 9:2738. doi: 10.3389/fpsyg.2018.02738 30687189 PMC6333704

[ref12] Cejudo A. B. López-Rojas C. Gómez-Ariza C. J. Bajo M. T. (2022). ERP correlates of prospective memory and Cue Focality in children. Brain Sci. 12:533. doi: 10.3390/brainsci12050533, PMID: 35624918 PMC9138550

[ref13] Cejudo A. B. McDaniel M. A. Bajo M. T. (2019b). Event versus activity-based cues and motivation in school-related prospective memory tasks. PLoS One 14:e0215845. doi: 10.1371/journal.pone.0215845, PMID: 31002710 PMC6474621

[ref14] Chasteen A. L. Park D. C. Schwarz N. (2001). Implementation intentions and facilitation of prospective memory. Psychol. Sci. 12, 457–461. doi: 10.1111/1467-9280.00385, PMID: 11760131

[ref15] Cheie L. Opriș A. M. Visu-Petra L. (2021). Remembering the future: age-related differences in schoolchildren’s prospective memory depend on the cognitive resources employed by the task. Cogn. Dev. 58:101048. doi: 10.1016/j.cogdev.2021.101048

[ref16] Chen Y. Lian R. Yang L. Liu J. Meng Y. (2017). Working memory load and reminder effect on event-based prospective memory of high-and low-achieving students in math. J. Learn. Disabil. 50, 602–608. doi: 10.1177/0022219416668322, PMID: 27608655

[ref17] Costa A. Perri R. Serra L. Barban F. Gatto I. Zabberoni S. . (2010). Prospective memory functioning in mild cognitive impairment. Neuropsychol. 24, 327–335. doi: 10.1037/a0018015 20438210

[ref18] Cottini M. Basso D. Palladino P. (2018). The role of declarative and procedural metamemory in event-based prospective memory in school-aged children. J. Exp. Child Psychol. 166, 17–33. doi: 10.1016/j.jecp.2017.08.002, PMID: 28858667

[ref19] Cottini M. Basso D. Palladino P. (2021a). Improving prospective memory in school-aged children: effects of future thinking and performance predictions. J. Exp. Child Psychol. 204:105065. doi: 10.1016/j.jecp.2020.105065, PMID: 33422737

[ref20] Cottini M. Basso D. Pieri A. Palladino P. (2021b). Metacognitive monitoring and control in Children’s prospective memory. J. Cogn. Dev. 22, 619–639. doi: 10.1080/15248372.2021.1916500

[ref21] Cottini M. Basso D. Saracini C. Palladino P. (2019). Performance predictions and postdictions in prospective memory of school-aged children. J. Exp. Child Psychol. 179, 38–55. doi: 10.1016/j.jecp.2018.10.008 30476694

[ref22] Cottini M. Meier B. (2020). Prospective memory monitoring and aftereffects of deactivated intentions across the lifespan. Cogn. Dev. 53:100844. doi: 10.1016/j.cogdev.2019.100844

[ref23] DeMarie D. Miller P. H. Ferron J. Cunningham W. R. (2004). Path analysis tests of theoretical models of children's memory performance. J. Cogn. Dev. 5, 461–492. doi: 10.1207/s15327647jcd0504_4

[ref24] Einstein G. O. Holland L. J. McDaniel M. A. Guynn M. J. (1992). Age-related deficits in prospective memory: the influence of task complexity. Psychol. Aging 7, 471–478. doi: 10.1037/0882-7974.7.3.471, PMID: 1388869

[ref25] Einstein G. O. McDaniel M. A. (1990). Normal aging and prospective memory. J. Exp. Psychol. Learn. Mem. Cogn. 16, 717–726. doi: 10.1037/0278-7393.16.4.717 2142956

[ref26] Ellis J. (1996). “Prospective memory or the realization of delayed intentions: A conceptual framework for research” in Prospective memory: theory and applications, eds. Brandimonte M. Einstein G. O. McDaniel A. (Lawrence Erlbaum Associates) 1–22.

[ref27] Ford R. M. Driscoll T. Shum D. Macaulay C. E. (2012). Executive and theory-of-mind contributions to event-based prospective memory in children: exploring the self-projection hypothesis. J. Exp. Child Psychol. 111, 468–489. doi: 10.1016/j.jecp.2011.10.006, PMID: 22169353

[ref28] Geurten M. Lejeune C. Meulemans T. (2016). Time’s up! Involvement of metamemory knowledge, executive functions, and time monitoring in children’s prospective memory performance. Child Neuropsychol. 22, 443–457. doi: 10.1080/09297049.2014.998642 25732049

[ref29] Godfrey M. Casnar C. Stolz E. Ailion A. Moore T. Gioia G. (2023). A review of procedural and declarative metamemory development across childhood. Child Neuropsychol. 29, 183–212. doi: 10.1080/09297049.2022.2055751, PMID: 35343879

[ref30] Guajardo N. R. Best D. L. (2000). Do preschoolers remember what to do? Incentive and external cues in prospective memory. Cogn. Dev. 15, 75–97. doi: 10.1016/S0885-2014(00)00016-2

[ref31] Guynn M. J. McDaniel M. A. Einstein G. O. (2001). Remembering to perform actions. A different type of memory? In memory for action: a distinct form of episodic memory? vol. 25. Oxford Academic.

[ref32] Han P. Han L. Bian Y. Tian Y. Xu M. Gao F. (2017). Influence of ongoing task difficulty and motivation level on children’s prospective memory in a Chinese sample. Front. Psychol. 8:89. doi: 10.3389/fpsyg.2017.00089 28203212 PMC5285343

[ref33] Hartwig J. Kretschmer-Trendowicz A. Helmert J. R. Jung M. L. Pannasch S. (2021). Revealing the dynamics of prospective memory processes in children with eye movements. Int. J. Psychophysiol. 160, 38–55. doi: 10.1016/j.ijpsycho.2020.12.005, PMID: 33387575

[ref34] Hashimoto T. Yokota S. Umeda S. Kawashima R. (2022). Dynamic functional connectivity associated with prospective memory success in children. Neuroimage 2:100144. doi: 10.1016/j.ynirp.2022.100144 PMC12172931 40567559

[ref35] Henry J. D. Terrett G. Altgassen M. Raponi-Saunders S. Ballhausen N. Schnitzspahn K. M. . (2014). A virtual week study of prospective memory function in autism spectrum disorders. J. Exp. Child Psychol. 127, 110–125. doi: 10.1016/j.jecp.2014.01.011, PMID: 24679459

[ref36] Hongwanishkul D. Happaney K. R. Lee W. S. C. Zelazo P. D. (2005). Assessment of hot and cool executive function in young children: age-related changes and individual differences. Dev. Neuropsychol. 28, 617–644. doi: 10.1207/s15326942dn2802_4, PMID: 16144430

[ref37] Hutchens R. L. Kinsella G. J. Ong B. Pike K. E. Parsons S. Storey E. . (2012). Knowledge and use of memory strategies in amnestic mild cognitive impairment. Psychol. Aging 27, 768–777. doi: 10.1037/a0026256 22122606

[ref38] Kelly A. J. Camden A. A. Williams M. C. Beran M. J. Perdue B. M. (2023). Habitual prospective memory in preschool children. PLoS One 18:e0293599. doi: 10.1371/journal.pone.0293599, PMID: 37906551 PMC10617692

[ref39] Kerns K. A. (2000). The CyberCruiser: an investigation of development of prospective memory in children. J. Int. Neuropsychol. Soc. 6, 62–70. doi: 10.1017/S1355617700611074, PMID: 10761368

[ref40] Kerr A. Zelazo P. D. (2004). Development of “hot” executive function: the children’s gambling task. Brain Cogn. 55, 148–157. doi: 10.1016/S0278-2626(03)00275-6, PMID: 15134849

[ref41] Khan A. Sharma N. K. Dixit S. (2008). Cognitive load and task condition in event-and time-based prospective memory: an experimental investigation. J. Psychol. 142, 517–532. doi: 10.3200/JRLP.142.5.517-532, PMID: 18959223

[ref42] Kidder D. P. Park D. C. Hertzog C. Morrell R. W. (1997). Prospective memory and aging: the effects of working memory and prospective memory task load. Aging Neuropsychol. Cognit. 4, 93–112. doi: 10.1080/13825589708256639

[ref43] Kliegel M. Jäger T. (2007). The effects of age and cue-action reminders on event-based prospective memory performance in preschoolers. Cogn. Dev. 22, 33–46. doi: 10.1016/j.cogdev.2006.08.003

[ref44] Kliegel M. Mackinlay R. Jäger T. (2008). Complex prospective memory: development across the lifespan and the role of task interruption. Dev. Psychol. 44, 612–617. doi: 10.1037/0012-1649.44.2.612, PMID: 18331148

[ref45] Kliegel M. Mahy C. E. V. Voigt B. Henry J. D. Rendell P. G. Aberle I. (2013). The development of prospective memory in young schoolchildren: the impact of ongoing task absorption, cue salience, and cue centrality. J. Exp. Child Psychol. 116, 792–810. doi: 10.1016/j.jecp.2013.07.012, PMID: 24056203

[ref46] Kliegel M. Martin M. McDaniel M. A. Einstein G. O. (2002). Complex prospective memory and executive control of working memory: a process model. Psychol. Test Assess. Model. 44, 303–318.

[ref47] Kliegel M. McDaniel M. A. Einstein G. O. (2000). Plan formation, retention, and execution in prospective memory: A new approach and age-related effects. Mem. Cogn. 28, 1041–1049. doi: 10.3758/BF03209352, PMID: 11105530

[ref48] Kmet L. M. Cook L. S. Lee R. C. (2004). Standard quality assessment criteria for evaluating primary research papers from a variety of fields. Alberta Heritage Foundation for Medical Research. doi: 10.7939/R37M04F16

[ref49] Kretschmer A. Voigt B. Friedrich S. Pfeiffer K. Kliegel M. (2014). Time-based prospective memory in young children—exploring executive functions as a developmental mechanism. Child Neuropsychol. 20, 662–676. doi: 10.1080/09297049.2013.841881, PMID: 24111941

[ref50] Kretschmer-Trendowicz A. Ellis J. A. Altgassen M. (2016). Effects of episodic future thinking and self-projection on children’s prospective memory performance. PLoS One 11:e0158366. doi: 10.1371/journal.pone.0158366, PMID: 27355645 PMC4927109

[ref51] Kretschmer-Trendowicz A. Kliegel M. Goschke T. Altgassen M. (2021). ‘If-then’ but when? Effects of implementation intentions on children’s and adolescents’ prospective memory. Cogn. Dev. 57:100998. doi: 10.1016/j.cogdev.2020.100998

[ref52] Kretschmer-Trendowicz A. Schnitzspahn K. M. Reuter L. Altgassen M. (2019). Episodic future thinking improves children’s prospective memory performance in a complex task setting with real life task demands. Psychol. Res. 83, 514–525. doi: 10.1007/s00426-017-0908-0 28861602

[ref53] Kvavilashvili L. Ford R. M. (2014). Metamemory prediction accuracy for simple prospective and retrospective memory tasks in 5-year-old children. J. Exp. Child Psychol. 127, 65–81. doi: 10.1016/j.jecp.2014.01.014, PMID: 24698432

[ref54] Kvavilashvili L. Messer D. J. Ebdon P. (2001). Prospective memory in children: the effects of age and task interruption. Dev. Psychol. 37, 418–430. doi: 10.1037/0012-1649.37.3.418 11370916

[ref55] Lachman M. E. Andreoletti C. (2006). Strategy use mediates the relationship between control beliefs and memory performance for middle-aged and older adults. J. Gerontol. Ser. B Psychol. Sci. Soc. Sci. 61, P88–P94. doi: 10.1093/geronb/61.2.P88, PMID: 16497959

[ref56] Lavis L. Mahy C. E. V. (2021). “I’ll remember everything no matter what!”: the role of metacognitive abilities in the development of young children’s prospective memory. J. Exp. Child Psychol. 207:105117. doi: 10.1016/j.jecp.2021.105117, PMID: 33676117

[ref57] Lehmann M. Hasselhorn M. (2007). Variable memory strategy use in children’s adaptive intratask learning behavior: developmental changes and working memory influences in free recall. Child Dev. 78, 1068–1082. doi: 10.1111/j.1467-8624.2007.01053.x 17650126

[ref58] Lejeune C. Catale C. Schmitz X. Quertemont E. Meulemans T. (2013). Age-related differences in perceptuomotor procedural learning in children. J. Exp. Child Psychol. 116, 157–168. doi: 10.1016/j.jecp.2013.05.001, PMID: 23773917

[ref59] Mahy C. E. V. Mohun H. Müller U. Moses L. J. (2016). The role of subvocal rehearsal in preschool children’s prospective memory. Cogn. Dev. 39, 189–196. doi: 10.1016/j.cogdev.2016.07.001

[ref60] Mahy C. E. V. Moses L. J. (2011). Executive functioning and prospective memory in young children. Cogn. Dev. 26, 269–281. doi: 10.1016/j.cogdev.2011.06.002

[ref61] Mahy C. E. V. Moses L. J. (2015). The effect of retention interval task difficulty on young children’s prospective memory: testing the intention monitoring hypothesis. J. Cogn. Dev. 16, 742–758. doi: 10.1080/15248372.2014.930742

[ref62] Mahy C. E. V. Moses L. J. Kliegel M. (2014a). The development of prospective memory in children: an executive framework. Dev. Rev. 34, 305–326. doi: 10.1016/j.dr.2014.08.001

[ref63] Mahy C. E. V. Moses L. J. Kliegel M. (2014b). The impact of age, ongoing task difficulty, and cue salience on preschoolers’ prospective memory performance: the role of executive function. J. Exp. Child Psychol. 127, 52–64. doi: 10.1016/j.jecp.2014.01.006 24613075

[ref64] Mahy C. E. V. Voigt B. Ballhausen N. Schnitzspahn K. Ellis J. Kliegel M. (2015). The impact of cognitive control on children’s goal monitoring in a time-based prospective memory task. Child Neuropsychol. 21, 823–839. doi: 10.1080/09297049.2014.967202, PMID: 25342074

[ref65] Mäntylä T. Carelli M. G. Forman H. (2007). Time monitoring and executive functioning in children and adults. J. Exp. Child Psychol. 96, 1–19. doi: 10.1016/j.jecp.2006.08.003 17030038

[ref66] McDaniel M. A. Glisky E. L. Guynn M. J. Routhieaux B. C. (1999). Prospective memory: a neuropsychological study. Neuropsychology 13, 103–110. doi: 10.1037/0894-4105.13.1.103 10067781

[ref67] McNamara D. S. Scott J. L. (2001). Working memory capacity and strategy use. Mem. Cogn. 29, 10–17. doi: 10.3758/BF03195736 11277453

[ref68] Meacham J. A. Colombo J. A. (1980). External retrieved cues facilitate prospective remembering in children. J. Educ. Res. 73, 299–301. doi: 10.1080/00220671.1980.10885254

[ref69] Mills G. N. Garbarino J. T. Raskin S. A. (2021). Assessing prospective memory in children using the memory for intentions screening test for youth (MISTY). Clin. Neuropsychol. 35, 643–659. doi: 10.1080/13854046.2019.1711198, PMID: 31933412

[ref70] Miyake A. Friedman N. P. Emerson M. J. Witzki A. H. Howerter A. Wager T. D. (2000). The unity and diversity of executive functions and their contributions to complex “frontal lobe” tasks: A latent variable analysis. Cogn. Psychol. 41, 49–100, PMID: 10945922 10.1006/cogp.1999.0734

[ref71] Norman D. A. Shallice T. (1986). Attention to action: Willed and automatic control of behavio. In Consciousness and self-regulation. Vol. 1, eds. Davidson R. J. Shallice T. (New York, NY: Springer Science+Business Media), 1–18.

[ref72] Raskin S. A. Buckheit C. Sherrod C. (2010). Memory for intentions screening test (MIST) [Database record]. APA PsycTests. doi: 10.1037/t74479-000

[ref73] Rendell P. G. Craik F. I. (2000). Virtual week and actual week: age-related differences in prospective memory. Appl. Cogn. Psychol. 14, S43–S62. doi: 10.1002/acp.770

[ref74] Rendell P. G. Vella M. J. Kliegel M. Terrett G. (2009). Effect of delay on children’s delay-execute prospective memory performance. Cogn. Dev. 24, 156–168. doi: 10.1016/j.cogdev.2008.12.002

[ref75] Ryder N. Kvavilashvili L. Ford R. (2022). Effects of incidental reminders on prospective memory in children. Dev. Psychol. 58, 890–901. doi: 10.1037/dev0001035, PMID: 35311306

[ref76] Schneider W. Lockl K. (2008). “Procedural metacognition in children: evidence for developmental trends” in Handbook of Metamemory and Memory, *1st Edn*. eds. Dunlosky J. Bjork R. A. (Psychology Press), 14, 391–409.

[ref77] Schneider W. Pressley M. (2013). Introduction to memory development during childhood and adolescence. *1st Edn*. New York, NY: Psychology Press.

[ref79] Shum D. Cross B. Ford R. Ownsworth T. (2008). A developmental investigation of prospective memory: effects of interruption. Child Neuropsychol. 14, 547–561. doi: 10.1080/09297040801947051, PMID: 18608222

[ref80] Simons J. S. Schölvinck M. L. Gilbert S. J. Frith C. D. Burgess P. W. (2006). Differential components of prospective memory?: evidence from fMRI. Neuropsychologia 44, 1388–1397. doi: 10.1016/j.neuropsychologia.2006.01.005, PMID: 16513147

[ref81] Ślusarczyk E. Niedźwieńska A. (2013). A naturalistic study of prospective memory in preschoolers: the role of task interruption and motivation. Cogn. Dev. 28, 179–192. doi: 10.1016/j.cogdev.2012.10.004

[ref82] Ślusarczyk E. Niedźwieńska A. Białecka-Pikul M. (2018). The first signs of prospective memory. Memory 26, 1385–1395. doi: 10.1080/09658211.2018.1483516 29869574

[ref83] Smith R. E. Bayen U. J. Martin C. (2010). The cognitive processes underlying event-based prospective memory in school-age children and young adults: a formal model-based study. Dev. Psychol. 46, 230–244. doi: 10.1037/a0017100, PMID: 20053020 PMC2856082

[ref84] Somerville S. C. Wellman H. M. Cultice J. C. (1983). Young children’s deliberate reminding. J. Genet. Psychol. 143, 87–96. doi: 10.1080/00221325.1983.10533537

[ref85] Szpakiewicz E. Stępień-Nycz M. (2024). How do the cognitive processes matter in the event-based preschoolers’ prospective memory? Front. Psychol. 15:1279144. doi: 10.3389/fpsyg.2024.1279144, PMID: 38699576 PMC11063366

[ref86] Terrett G. Horner K. White R. Henry J. D. Kliegel M. Labuschagne I. . (2019). The relationship between episodic future thinking and prospective memory in middle childhood: mechanisms depend on task type. J. Exp. Child Psychol. 219, 92–99. doi: 10.1027/2151-2604/a000053, PMID: 30388484

[ref87] Voigt B. Aberle I. Schönfeld J. Kliegel M. (2015). Time-based prospective memory in schoolchildren. Z. Psychol. 178, 198–213. doi: 10.1016/j.jecp.2018.10.003

[ref88] Voigt B. Mahy C. E. V. Ellis J. Schnitzspahn K. Krause I. Altgassen M. . (2014). The development of time-based prospective memory in childhood: the role of working memory updating. Dev. Psychol. 50, 2393–2404. doi: 10.1037/a0037491, PMID: 25111770

[ref89] Walsh S. J. Martin G. M. Courage M. L. (2014). The development of prospective memory in preschool children using naturalistic tasks. J. Exp. Child Psychol. 127, 8–23. doi: 10.1016/j.jecp.2013.10.003 24290293

[ref90] Wang L. Kliegel M. Liu W. Yang Z. (2008). Prospective memory performance in preschoolers: inhibitory control matters. Eur. J. Dev. Psychol. 5, 289–302. doi: 10.1080/17405620600778161

[ref91] Wang Y. Ren Z. Yue Y. Zheng X. Zhang X. Wang L. (2024). The effect of time monitoring on the development of time-based prospective memory among children aged 7–11 years old. Behav. Sci. 14:233. doi: 10.3390/bs14030233, PMID: 38540536 PMC10967771

[ref92] Ward H. Shum D. McKinlay L. Baker S. Wallace G. (2007). Prospective memory and pediatric traumatic brain injury: effects of cognitive demand. Child Neuropsychol. 13, 219–239. doi: 10.1080/09297040600910003, PMID: 17453831

[ref78] Weinert F. E. Schneider W. (Eds.) (1995). Memory performance and competencies: Issues in growth and development. Mahwah, NJ, Hove, UK: Lawrence Erlbaum Associates, Inc.

[ref93] Winograd E. (1988). Some observations on prospective remembering. In Practical aspects of memory: Current research and issues, Vol. 1. Memory in everyday life. eds. Gruneberg M. M. Morris P. E. Sykes R. N. (John Wiley & Sons, Inc.), 348–353.

[ref94] Yang T. Chan R. C. K. Shum D. (2011). The development of prospective memory in typically developing children. Neuropsychology 25, 342–352. doi: 10.1037/a0022239 21443342

[ref95] Yang T. X. Zhang S. Y. Wang Y. Su X. M. Yuan C. W. Lui S. S. . (2023). The effect of implementation intentions on event-, time-, and activity-based prospective memory in typically developing children. Int. J. Behav. Dev. 47, 146–157. doi: 10.1177/01650254221146420

[ref96] Zhang X. Zuber S. Liu S. Kliegel M. Wang L. (2017). The effects of task instructor status on prospective memory performance in preschoolers. Eur. J. Dev. Psychol. 14, 102–117. doi: 10.1080/17405629.2016.1165660

[ref97] Zhang X. Zuber S. Zhang J. Ihle A. Kliegel M. Wang L. (2019). The influence of ongoing task absorption on preschoolers’ prospective memory with peripheral cues. J. Cogn. Psychol. 31, 522–532. doi: 10.1080/20445911.2019.1646747

[ref98] Zimmermann T. D. Meier B. (2006). The rise and decline of prospective memory performance across the lifespan. Q. J. Exp. Psychol. 59, 2040–2046. doi: 10.1080/17470210600917835, PMID: 17095485

[ref99] Zinke K. Altgassen M. Mackinlay R. J. Rizzo P. Drechsler R. Kliegel M. (2010). Time-based prospective memory performance and time-monitoring in children with ADHD. Child Neuropsychol. 16, 338–349. doi: 10.1080/09297041003631451, PMID: 20336559

[ref100] Zöllig J. West R. Martin M. Altgassen M. Lemke U. Kliegel M. (2007). Neural correlates of prospective memory across the lifespan. Neuropsychologia 45, 3299–3314. doi: 10.1016/j.neuropsychologia.2007.06.010 17675111

[ref101] Zuber S. Mahy C. E. V. Kliegel M. (2019). How executive functions are associated with event-based and time-based prospective memory during childhood. Cogn. Dev. 50, 66–79. doi: 10.1016/j.cogdev.2019.03.001

